# The Use of Telerehabilitation to Improve Movement-Related Outcomes and Quality of Life for Individuals With Parkinson Disease: Pilot Randomized Controlled Trial

**DOI:** 10.2196/54599

**Published:** 2024-07-31

**Authors:** Joshua K. Johnson, Jason K Longhurst, Michael Gevertzman, Corey Jefferson, Susan M Linder, Francois Bethoux, Mary Stilphen

**Affiliations:** 1 Rehabilitation and Sports Therapy Neurological Institute Cleveland Clinic Cleveland, OH United States; 2 Center for Value-Based Care Research Cleveland Clinic Cleveland, OH United States; 3 Department of Physical Therapy and Athletic Training Saint Louis University Saint Louis, MO United States; 4 Department of Physical Medicine and Rehabilitation Neurological Institute Cleveland Clinic Cleveland, OH United States

**Keywords:** rehabilitation, physical therapy, PT, physiotherapy, telehealth, Parkinson disease, tele-rehabilitation, telerehabilitation, TR, exercise, physical activity, exercise therapy, tele-health, mHealth, mobile health, app, apps, application, applications, digital health, smartphone, smartphones, Parkinson’s disease, Parkinson, Parkinsons, Parkinsonism, PD

## Abstract

**Background:**

Individuals with Parkinson disease (PD) can improve their overall mobility and participation in daily activities as they engage in frequent exercise. Despite the need for individually tailored exercises, persons with PD often face barriers to accessing physical rehabilitation professionals who can provide them. Telerehabilitation (TR) may facilitate access to necessary and individually tailored rehabilitation for individuals with PD.

**Objective:**

The purpose of this study was to assess the feasibility of TR for individuals with PD and explore clinical outcomes compared to in-person care.

**Methods:**

This was a pilot randomized controlled trial conducted at 2 outpatient neurorehabilitation clinics with 3 study groups: clinic+TR, TR-only, and usual care (UC). TR was administered using a web-based application with a mobile app option. One-hour interventions were performed weekly for 4 weeks, in-person for the clinic+TR and UC groups and virtually for the TR-only group. Home exercises were provided on paper for the UC group and via the web-based platform for the clinic+TR and TR-only groups. Feasibility was assessed by recruitment and retention success and patient and therapist satisfaction, as rated in surveys. Clinical outcomes were explored using performance and patient-reported measures in between- and within-group analyses.

**Results:**

Of 389 patients screened, 68 (17.5%) met eligibility criteria, and 20 (29.4% of those eligible) were enrolled (clinic+TR, n=6; TR-only, n=6; and UC, n=8). One patient (TR-only) was withdrawn due to a non–study-related injurious fall. Regardless of group allocation, both patients and therapists generally rated the mode of care delivery as “good” or “very good” across all constructs assessed, including overall satisfaction and safety. In the analysis of all groups, there were no differences in clinical outcomes at the discharge visit. Within-group differences (from baseline to discharge) were also generally not significant except in the UC group (faster 5-time sit-to-stand time and higher mini balance evaluation systems test balance score) and clinic+TR group (higher mini balance evaluation systems test balance score).

**Conclusions:**

High satisfaction amongst patients and clinicians regardless of group, combined with nonsignificant between-group differences in clinical outcomes, suggest that TR is feasible for individuals with PD in early-moderate stages. Future trials with a larger sample are necessary to test clinical effectiveness. As larger trials enroll patients with diverse characteristics (eg, in terms of age, disease progression, caregiver support, technology access and capacity, etc), they could begin to identify opportunities for matching patients to the optimal utilization of TR as part of the therapy episode.

**Trial Registration:**

ClinicalTrials.gov NCT06246747; https://clinicaltrials.gov/study/NCT06246747

## Introduction

Individuals with Parkinson disease (PD) can improve their overall mobility and participation in daily activities as they engage in frequent exercise [1-6]. Further, exercise may provide a neuroprotective effect for these individuals, thereby limiting the progression of the disease [7]. As in other patient populations, individuals with PD demonstrate the greatest benefit from an exercise program that is designed to meet their specific needs [8,9].

Despite the need for individually tailored exercise programs, persons with PD often face barriers to accessing physical rehabilitation professionals who can provide them. Patients may live too far from a specialized clinic, be unable to secure adequate transportation to a clinic, or face structural barriers if the clinic’s physical environment is not conducive to allowing safe movement throughout [10,11]. Additionally, system-level barriers like limitations in the number of adequately trained rehabilitation clinicians and poorly aligned reimbursement policies may limit the frequency with which persons with PD can receive the specified exercise interventions that may be of greatest benefit to them [12,13].

Medical care—“telemedicine”—via phone or video calls is preferred by many patients with PD and their often overburdened caregivers as it is more convenient and less costly [11]. Its extension to rehabilitation—“telerehabilitation”—can address many of the barriers faced by those with PD to receive specialized care [14,15]. Prescribing at-home exercises is a common practice of physical therapists; however, generic home exercise programs that do not incorporate disease- and patient-specific components are not beneficial [8]. Customized programs that incorporate PD-specific exercises that are individually tailored to address an individual’s symptoms and goals, however, may be feasible and effective [16,17].

The primary aim of this pilot randomized controlled trial was to assess the feasibility of telerehabilitation (TR) for patients with PD by examining recruitment and retention success and exploring perceptions of satisfaction among patients and the physical therapists that provided their interventions. We also conducted a preliminary exploratory examination of the clinical effectiveness of TR for persons with PD.

## Methods

### Setting

The study was conducted at 2 Cleveland Clinic outpatient rehabilitation clinics in Cleveland, Ohio, and Las Vegas, Nevada. Both outpatient clinics specialize in rehabilitation for degenerative neurological diseases. At any given time, 2 full-time physical therapists at each site participated in study procedures. A total of 5 physical therapists engaged in study procedures and interventions over the duration of the study.

### Study Design

This was an unblinded, randomized controlled trial. The randomization allocation sequence was generated by the principal investigator. Randomization was concealed and completed by a study coordinator using the REDCap (Research Electronic Data Capture; Vanderbilt University) randomization module [18]. Patients were 1:1 block randomized by site into 1 of 3 groups: a clinic+TR group, a TR-only group, and a usual care (UC) control group. Each participant was in the study for 5 weeks, which included an initial evaluation, 4 weeks of therapy, and a discharge visit. For all 4 therapy weeks, those in the clinic+TR group participated in physical therapy once per week in the clinic and in a daily therapist-prescribed home exercise program using the TR platform. Those in the TR-only group also participated in a daily therapist-prescribed home exercise program using the platform, but with once-weekly web-based visits (via video conference calls) taking the place of clinic visits. Those in the UC group participated in physical therapy once per week in the clinic and in a daily therapist-prescribed home exercise program provided on a printed paper handout.

### Participant Eligibility and Recruitment

Patients were recruited between February 1, 2020, and December 15, 2022. Initially, patients were identified for prescreening if they had a diagnosis of PD for any duration and were scheduled for a new episode of physical therapy. Prescreening included a chart review by a study coordinator. The exclusion criteria were as follows: any outpatient physical therapy episode in the preceding 5 months, documented diagnosis of another progressive neurological disease, history of epilepsy, moderate-to-severe cognitive impairment, hemiparesis or hemiplegia, and current complaints of dizziness. Though not an a priori criterion, some patients could not be scheduled for study participation due to a lack of availability on the schedules of participating therapists.

Those meeting eligibility criteria were contacted in writing (via a mailed letter or an electronic health record portal) and then via the phone by a coordinator 2-4 weeks prior to their initial physical therapy visit. At the prescreen phone call, patients were excluded if they were not reached, reported not having a reliable caregiver or internet connection in their home, or declined to participate.

After chart review and phone screening, those interested in participation completed the informed consent process in-person administered by a study coordinator and further screened for eligibility by the evaluating physical therapist immediately prior to their initial physical therapy visit. Final eligibility was determined by completion of the timed-up-and-go test (TUG) in <15 seconds, a 10-meter gait speed of >0.8 m/s, and a subjective determination of safety by the evaluating physical therapist.

### Eligibility and Recruitment Amendments

The protocol was amended significantly twice for the sake of encouraging enrollment, which was slowed due to the COVID-19 pandemic and the slow return of patients with PD to outpatient rehabilitation clinics. The first amendment (in November 2020) changed the criterion for the timing of any recent outpatient physical therapy episode from 5 months to 6 weeks. The second amendment (April 2022) eliminated the criterion related to recent outpatient physical therapy episodes, meaning that patients could be identified for prescreening even while participating in an ongoing physical therapy care episode. After the second amendment, the treating physical therapist made the patient aware of their potential study eligibility, which was confirmed by a study coordinator via chart review and a phone call. Those interested in participation were consented to in-person by the study coordinator and further screened for eligibility by the physical therapist immediately prior to a reassessment visit (occurring approximately 30 days after the initial evaluation).

### Recruitment Targets

The initial recruitment target was 30 patients. Our recruitment estimate was based on the participation of 2 physical therapists at each study site, an overall volume of 8 patients per month presenting to each clinic for a new evaluation for PD-related movement impairments and who would be expected to meet this study’s inclusion and exclusion criteria, and an estimated capture rate of 15%. We expected to meet our target recruitment in 15 months. The entire duration of the study, from training through reporting, was expected to be 24 months.

Due to slowed enrollment throughout the COVID-19 pandemic, the target was decreased to 20 patients in conjunction with the April 2022 amendment described earlier.

### Intervention

The primary intervention of interest in this study was the delivery of physical therapist interventions using a TR platform (WizeCare Technologies). Patients completed TR on a tablet (Lenovo Yoga Tab 3, Model YT3-X50F, OS Android 6.0) provided by WizeCare, their own tablet, their own laptop or desktop computer, or their own cell phone. If by tablet or cell phone, the platform was accessed using a mobile app. If by computer, the platform was accessed via a web browser.

The WizeCare TR platform enables remote rehabilitation training and monitoring using a customized combination of video conferencing and libraries of prerecorded videos of specific exercises. The videos were combined manually to create customized exercise prescriptions for patients that varied in content and duration. Immediately prior to the study, a series of videos of common PD-specific exercises were produced and stored in a specific library on the platform. Physical therapists built customized and individualized home exercise programs primarily from this library, though videos from any library were available for use. The platform also featured an option for live video calls between patients and therapists, which enabled ongoing communication and re-evaluation for adjustment of the rehabilitation plan as needed. No updates were incorporated into the TR platform during the course of this trial.

### Baseline Variables

Baseline demographic variables were age and sex. Baseline clinical variables were years since PD diagnosis, the modified Hoehn and Yahr scale, and the Movement Disorder Society–sponsored Unified Parkinson’s Disease Rating Scale Part III (MDS-UPDRS III) score. Hoehn and Yahr stages range from 1 (unilateral involvement only) to 5 (wheelchair-bound or bedridden unless aided) [19], with the modified scale including stages 1.5 and 2.5 to account for intermediate phases of disease progression. The MDS-UPDRS III is a clinician-rated instrument of observable motor-related PD symptoms (eg, rigidity, tremor, voluntary hand movements, gait, etc); higher MDS-UPDRS III scores indicate greater symptom severity [20].

### Outcomes

As a pilot study, the primary outcomes of interest were recruitment success (number eligible and number enrolled), the proportion of patients who withdrew from the study for any reason, and satisfaction among patients and therapists. For all groups, we assessed satisfaction with the mode of care delivery using surveys, which were administered to the patient and the physical therapist at the in-clinic discharge visit via the REDCap platform [18]. Participating physical therapists responded after each care episode, considering the mode of delivery for that specific patient. Each survey assessed 8 constructs; 6 satisfaction items were rated on a 5-point Likert scale (“very poor,” “poor,” “fair,” “good,” or “very good”), 1 item pertaining to safety used a 0 (“very unsafe”) to 100 (“very safe”) rating, and 1 item asked how likely the respondent would be to recommend their mode of delivery (for patient respondents) or use the same mode of delivery for a future similar patient (for therapist respondents). A final, open-ended question sought any other written feedback. The surveys were designed by the principal investigator and then pilot-tested by nonclinical members of the study team (since the clinical members of the study team would be completing the surveys as part of the study). The survey questions are presented in Multimedia Appendix 1.

Clinical measures were assessed at baseline by a study coordinator and at 5 weeks by the treating therapist using instruments that are reliable, valid, and sensitive to change in patients with PD. These included the TUG test [21,22]. TUG cognitive task assessment [23], the 5-time sit-to-stand (5STS) test [24,25], the 10-meter walk test [21], the 6-minute walk test (6MWT) [26], and the mini balance evaluation systems test (Mini-BESTest) [27]. Additionally, patients’ self-assessment of function was collected using the Parkinson’s Disease Questionnaire (PDQ-39) [28] and the Activities-specific Balance Confidence Scale (ABC) [22], both of which have been validated for use by patients with PD. The PDQ-39 summary index was used, which scales scores from 0 to 100, with higher scores indicating greater difficulty with tasks. Higher ABC scores indicate greater confidence.

### Patient Safety

At each interaction with the treating physical therapist—in clinic or via the TR platform—subjective feedback was provided verbally by the patient regarding the safety of the exercises he or she completed at home. This is consistent with current standard practice. Any reported fall or other adverse event, whether in conjunction with the exercise program or otherwise, was reviewed by the study team (the 4 treating physical therapists and the principal investigator). These were recorded, characterized as related or unrelated to the study intervention, and reported to the Institutional Review Board as required.

Additionally, the WizeCare platform enabled a safety assessment with each session in which a patient interacted with the system, including their unsupervised home exercise sessions. At the conclusion of each session on the platform, patients were asked, “Did you feel safe during this session?” If they answered “No,” the system sent an automated message to the treating physical therapist. The system also provided an automated response to the patient to, “Please stop participating in the exercise program and call your treating physical therapist.” Initiated by either the physical therapist or the patient within 24 hours of the reported lack of safety, a conversation between them allowed the patient to express any safety concerns. Any such events were recorded as adverse events, with subsequent steps as outlined earlier. These patient self-reports of safety were included among exploratory outcomes.

### Statistical Analysis

Patient characteristics and baseline measurements of each outcome were described by group. Within-group and between-group differences were compared. For normally distributed continuous variables (MDS-UPDRS III score, TUG time, TUG-Cognitive time, 10-meter walk test, 6MWT distance, Mini-BESTest score, and ABC), an ANOVA was used to compare all 3 groups, and an independent 2-tailed *t* test was used to compare group pairs. For continuous variables not normally distributed (age, years since PD diagnosis, Hoehn & Yahr stage, PDQ-39, and 5STS), the Kruskal-Wallis test was used to compare all 3 groups, and the Wilcoxon rank-sum test was used to compare group pairs. Between-group baseline differences in sex were compared using Fisher exact tests.

For the primary analysis of feasibility, Fisher exact tests were used to compare patient and physical therapist responses to the 6 survey items of care delivery satisfaction and the single question assessing recommendations for future care. The rating of safety was compared using the Kruskal-Wallis test.

The effect on clinical outcomes was exploratory. With so few observations per group in this pilot study, we acknowledged that the power of these statistical tests is limited, meaning they may have difficulty detecting true differences within and between groups, especially if the differences are small. The between-group differences in discharge clinical outcomes were compared using ANOVA for normally distributed data (TUG time, TUG-Cognitive time, 10-meter walk test, 6MWT distance, Mini-BESTest, and ABC) or Kruskal-Wallis tests for skewed data (PDQ-39 and 5STS). In addition, clinical outcomes between pairs of groups were compared using an independent *t* test or Kruskal-Wallis test. Within-group differences were compared using a dependent *t* test for normally distributed data and a Wilcoxon matched-pairs signed-rank test for skewed data. For all tests, α was set to 0.05.

### Ethical Considerations

This study, including a valid primary informed consent process, was approved by the Cleveland Clinic Institutional Review Board (#19-544). Only the necessary study personnel were given access to the electronic study data in a private instance of REDCap. The study was retrospectively registered at ClinicalTrials.gov (NCT06246747) since prospective registration was not deemed essential for this pilot study. Volunteer participants were not compensated for their participation, a point that was described during the informed consent process. Generative AI was not used at any stage of the study’s design, conduct, analysis, or reporting.

## Results

**Figure 1:**
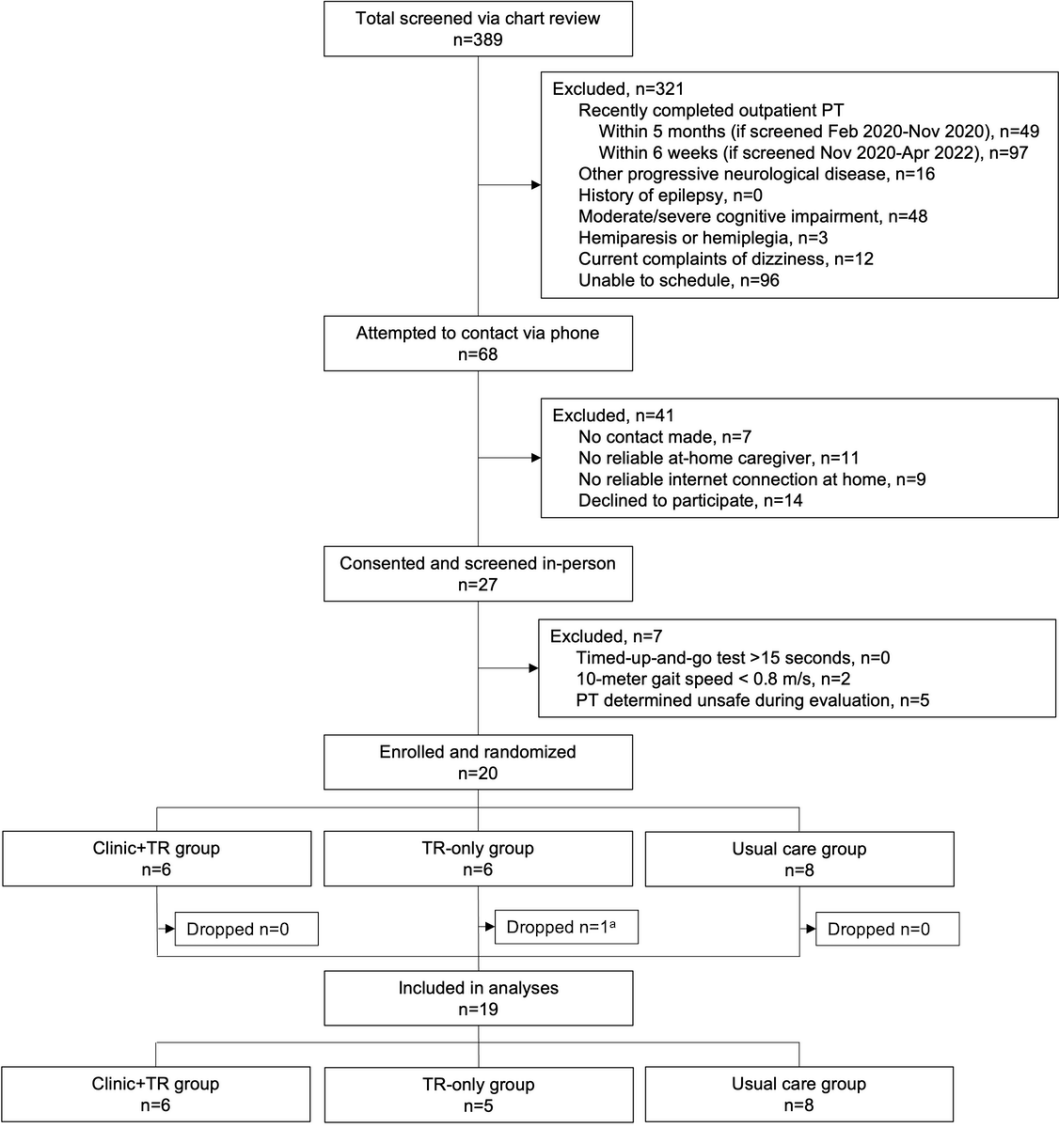
CONSORT (Consolidated Standards of Reporting Trials) flow diagram. PT: _; TR: telerehabilitation.

In sum, 389 patients were screened for potential inclusion. Of these, 68 (17.5%) met inclusion criteria; 27 (39.7% of those eligible) were consented to and screened in person (see the CONSORT [Consolidated Standards of Reporting Trials] diagram in Figure 1 [* indicates a non-study-related injurious fall]). After 7 patients were excluded at the in-person screening, 20 were enrolled and randomized to the clinic+TR group (n=6), the TR-only group (n=6), and the UC group (n=8). One patient (TR-only group) was withdrawn from the study after a non–study-related injurious fall that resulted in hospitalization. Two other patients (both in UC) reported non–study-related, noninjurious falls and remained in the study. There were no instances in which patients reported feeling unsafe after a TR session. Of the 19 who completed the study, 12 were enrolled in Las Vegas, and 7 were enrolled in Cleveland. There were 7 enrolled in a new episode of physical therapy, while 12 were enrolled as part of an established episode. A total of 5 physical therapists (2 in Las Vegas and 3 in Cleveland) provided the interventions, with each episode managed by only 1 physical therapist.

Baseline participant characteristics are shown in Table 1. There were no statistical differences between groups; however, potentially meaningful clinical differences were noted in years since PD diagnosis (median 5 (IQR 1-8) for the clinic+TR group compared to median 2 (IQR 0-3) for the TR-only and UC groups). Similarly, other baseline markers (MDS-UPDRS III score, Hoehn & Yahr stage, PDQ summary index, and ABC score) suggested advanced disease progression among those in the clinic+TR group compared to other groups.

**Figure 2:**
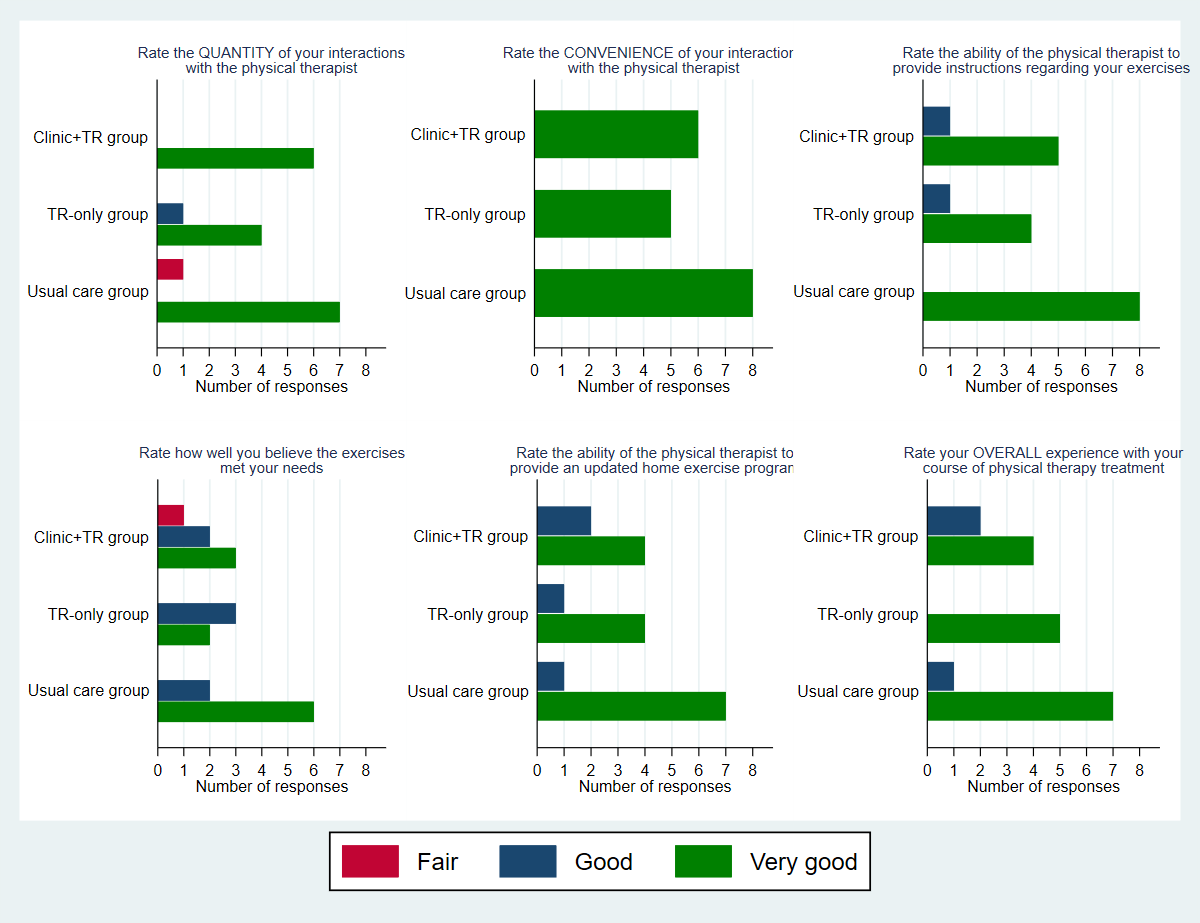
Patient ratings of satisfaction compared across 3 study groups. TR: telerehabilitation.

As shown in Figure 2, patients in all groups generally rated their care delivery as “good” or “very good” across all constructs assessed, including their overall satisfaction. No differences were statistically significant. However, fewer patients in both the clinic+TR and TR-only groups (50%, n=3 and 40%, n=2, respectively) gave a rating of “very good” than in the usual care group (75%, n=6) when asked if the prescribed exercises met their needs. Patients’ rating of safety was high across all groups (median 81.5, IQR 72-96 for clinic+TR; median 100, IQR 100-100 for TR-only; and median 98, IQR 82.5-100 for usual care); these ratings did significantly differ between groups (*P*=.049). Patients in all groups generally said they would “very likely” recommend their course of physical therapy for another patient with PD; exceptions were 1 patient in the clinic+TR group reporting “somewhat unlikely,” 1 patient in the TR-only group reporting “very unlikely,” and 1 patient in each of the TR-only group and usual care group reporting “somewhat likely.”

**Table 1:** Baseline patient characteristics for the full sample and across 3 study groups.

	Total (N=19)	Clinic+TR^a^ (n=6)	TR-only (n=5)	Usual care (n=8)	*P* values							
						Overall		Clinic+TR vs TR-only)		TR-only vs usual care		Clinic+TR vs usual care
Age (years), median (IQR)	73 (68-77)	74 (72-77)	74 (68-76)	68 (64-75)	.45		.78		.33		.27	
Gender, n (%)	12 (63.2)	2 (33.3)	3 (60.0)	7 (87.5)	.14		.57		.51		.09	
Years since PD^b^ diagnosis, median (IQR)	2 (0-7)	5 (1-8)	2 (0-3)	2 (0-3)	.47		.41		.82		.24	
MDS-UPDRS III^c^ score, mean (SD)	15.4 (8.8)	18.2 (11.1)	16.8 (5.9)	11.8 (7.7)	.45		.82		.30		.27	
Hoehn & Yahr stage, median (IQR)	1.5 (1.5-2.5)	2.25 (1.5-3.0)	1.5 (1.25-1.75)	1.5 (1.5-2.25)	.21		.10		.47		.22	
PDQ-39^d^ summary index, median (IQR)	43.6 (20.2-66.7)	53.4 (20.2-70.4)	40.7 (28.8-74.4)	42.5 (17.0-60.6)	.92		.81		.73		.77	
TUG^e^ (seconds), mean (SD)	8.7 (2.2)	9.5 (2.0)	8.5 (3.0)	8.3 (1.7)	.62		.53		.92		.29	
TUG-Cognitive (seconds), mean (SD)	11.1 (4.7)	12.4 (7.0)	10.5 (4.9)	10.4 (2.3)	.72		.63		.94		.46	
5-time STS^f^ (seconds), median (IQR)	10.4 (8.6-12.3)	11.0 (8.6-13.1)	11.4 (10.2-12.3)	10.1 (9.2-12.8)	.86		.86		.71		.61	
10-meter walk test (m/second), mean (SD)	1.2 (0.2)	1.1 (0.2)	1.2 (0.3)	1.2 (0.2)	.34		.26		.99		.15	
6MWT^g^ (ft), mean (SD)	1222.1 (515.5)	1040.7 (530.3)	1476.2 (353.2)	1199.3 (579.9)	.39		.15		.36		.61	
Mini-BESTest^h^ score, mean (SD)	20.6 (3.0)	19.8 (2.3)	21.8 (3.4)	20.5 (3.3)	.57		.29		.51		.68	
ABC^i^ score, mean (SD)	73.5 (19.0)	61.7 (24.2)	83.4 (12.9)	76.0 (14.8)	.15		.11		.38		.19	

^a^TR: telerehabilitation.

^b^PD: Parkinson disease.

^c^MDS-UPDRS: Movement Disorder Society-sponsored Unified Parkinson’s Disease Rating Scale.

^d^PDQ: Parkinson’s Disease Questionnaire.

^e^TUG: timed up-and-go.

^f^STS: sit-to-stand.

^g^MWT: minute walk test.

^h^BESTest: balance evaluation systems test.

^i^ABC: Activities-Specific Balance Confidence Scale.

**Figure 3:**
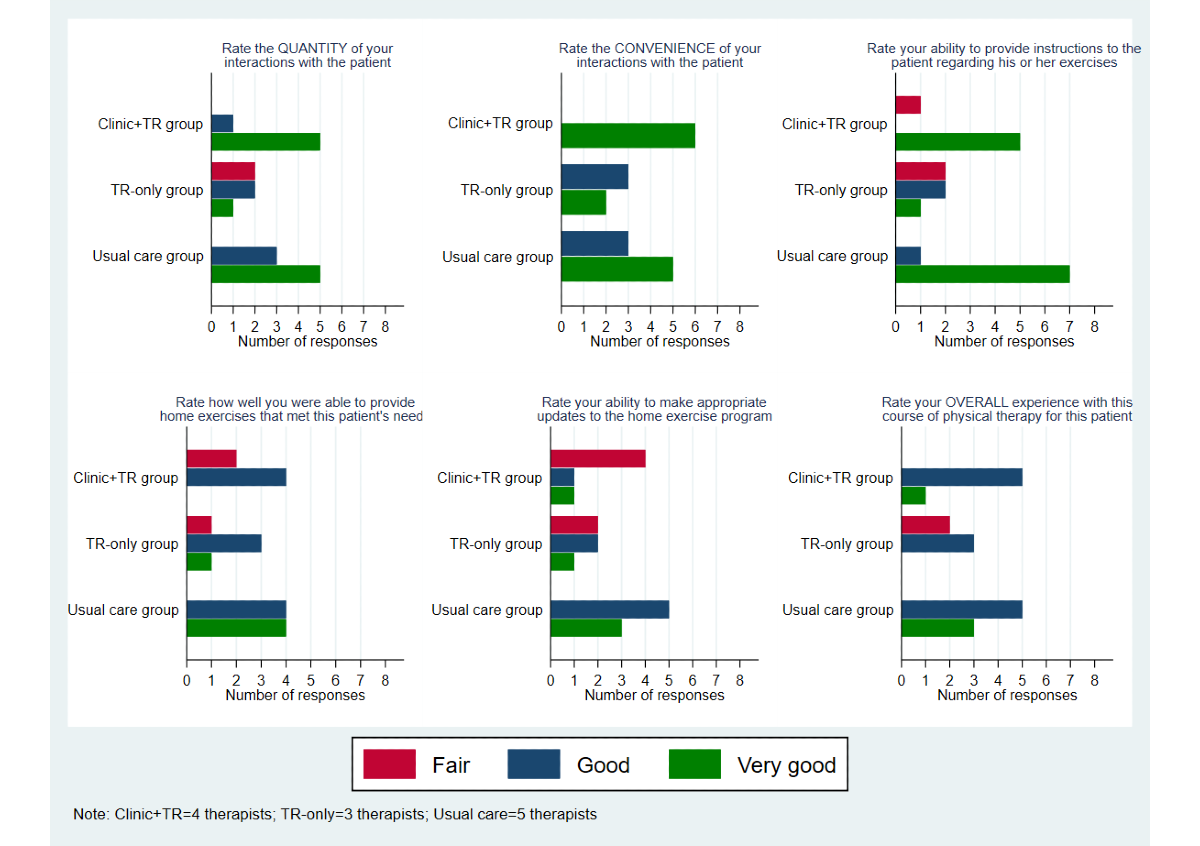
Therapist ratings of satisfaction compared across 3 study groups. TR: telerehabilitation.

Among the 5 physical therapists who participated as care providers, all 5 treated patients in the usual care group, 4 treated patients in the clinic+TR group, and 3 treated patients in the TR-only group. In rating their experience of the provision of care for all 19 patients who completed the study, the therapists’ responses were varied (Figure 3). Between groups, there was a statistical difference (*P*=.03) in therapists’ ratings of their ability to provide instructions to patients regarding prescribed exercises. Fewer therapists rated “Very good” for the TR-only group (20%, n=1) than for the usual care group (87.5%, n=7). No other differences in ratings were statistically significant. Therapists’ rating of safety for patients was high across all groups (median 91.5, IQR 71-100 for clinic+TR; median 85, IQR 82-88 for TR-only; and median 90, IQR 81-99 for usual care); these ratings did not differ significantly between groups (*P*=.81). Therapists’ responses were varied in regards to the likelihood that they would recommend a similar course of treatment for a patient with PD presenting with similar characteristics. Though not statistically significant (*P*=.08), the ratings for “Very likely” were 66.7% (n=4) for the clinic+TR group, 0% for the TR-only group, and 62.5% (n=5) for the usual care group.

Between all 3 groups, there were no statistically significant differences in clinical outcomes at the conclusion of the physical therapy episode. In pair-wise group comparisons, there was a difference in final ABC between those in the clinic+TR group (mean 57.7, SD 21.4) and the usual care group (mean 79.4, SD 11.4; *P*=.03). However, there was no within-group difference for the ABC in any group. We observed statistically significant improvements from baseline to final assessment in the usual care group for the 5STS time (median 10.1, IQR 9.2-12.8 seconds at baseline and median 9.2, IQR 8.8-11.3 seconds at final; *P*=.04) and the MiniBESTest score (mean 20.5, SD 3.3 at baseline and 23.0, SD 2.4 at final; *P*<.01). The clinic+TR group also improved in the MiniBESTest score from baseline to final (mean 19.8, SD 2.3 at baseline and 22.2, SD 2.2 at final; *P*=.02). Between-group comparisons are provided in Table 2; within-group comparisons are provided in Table 3.

Multimedia Appendix 2 includes a joint display that describes patient characteristics alongside patients’ and therapists’ recommendations for the same course of physical therapy for other patients with PD and relevant comments from patients and therapists.

**Table 2:** Between-group clinical outcomes at discharge.

Outcome	Clinic+TR^a^ (n=6)	TR-only (n=5)	Usual care (n=8)		*P* values			
				Overall		Clinic+TR vs TR-only	TR-only vs usual care	Clinic+TR vs usual care
PDQ-39^b^ Summary Index, median (IQR)	63.7 (28.1-77.0)	50.3 (30.1-94.0)	28.3 (17.3-37.2)		.33	.99	.24	.20
TUG^c^ (seconds), mean (SD)	8.7 (1.3)	8.8 (4.6)	8.3 (1.0)		.92	.98	.77	.49
TUG-Cognitive (seconds), mean (SD)	9.2 (1.6)	9.6 (5.9)	9.4 (1.0)		.98	.87	.93	.77
5-time STS^d^ (seconds), median (IQR)	10.8 (8.9-13.7)	11.7 (7.3-13.4)	9.2 (8.8-11.3)		.77	.85	.77	.44
10-meter walk test (m/second), mean (SD)	1.1 (0.1)	1.2 (0.4)	1.7 (1.1)		.33	.48	.38	.22
6MWT^e^ (ft), mean (SD)	1367.0 (137.1)	1491.5 (379.6)	1406.9 (416.5)		.83	.47	.72	.83
Mini-BESTest^f^ score, mean (SD)	22.2 (2.2)	23.6 (3.1)	23.0 (2.4)		.65	.40	.70	.52
ABC^g^ score, mean (SD)	57.7 (21.4)	70.8 (20.8)	79.4 (11.4)		.10	.33	.35	.03

^a^TR: telerehabilitation.

^b^PDQ: Parkinson’s Disease Questionnaire.

^c^TUG: timed up-and-go.

^d^STS: sit-to-stand.

^e^MWT: minute walk test.

^f^BESTest: balance evaluation systems test.

^g^ABC: Activities-Specific Balance Confidence Scale.

**Table 3:** Within-group clinical outcomes (baseline to discharge).

Outcome	Clinic+TR^a^ group (n=6)				TR-only group (n=5)				Usual care group (n=8)		
	Baseline	Discharge	*P* value	Baseline		Discharge	*P* value	Baseline		Discharge	*P* value
PDQ-39^b^ Summary Index, median (IQR)	53.44 (20.16-70.42)	63.70 (28.13-76.98)	.10	40.70 (28.78-74.35)		50.31 (30.10-94.01)	.99	42.45 (17.01-60.60)		28.33 (17.32-37.19)	.12
TUG^c^ (seconds), mean (SD)	9.45 (2.01)	8.70 (1.31)	.28	8.46 (3.03)		8.76 (4.61)	.83	8.33 (1.74)		8.27 (1.01)	.85
TUG-Cognitive (seconds), mean (SD)	12.40 (7.04)	9.15 (1.58)	.33	10.53 (4.86)		9.56 (5.90)	.64	10.39 (2.33)		9.36 (1.00)	.16
5-time STS^d^ (seconds), median (IQR)	10.99 (8.60-13.10)	10.84 (8.86-13.70)	.84	11.38 (10.16-12.28)		11.65 (7.34-13.35)	.89	10.08 (9.20-12.80)		9.23 (8.76-11.28)	.04
10-meter walk test (m/second), mean (SD)	1.05 (0.15)	1.11 (0.11)	.27	1.20 (0.25)		1.22 (0.35)	.84	1.20 (0.19)		1.66 (1.05)	.21
6MWT^e^ (ft), mean (SD)	1040.67 (530.28)	1367.00 (137.06)	.17	1476.20 (353.17)		1491.50 (379.55)	.88	1199.26 (579.85)		1406.88 (416.53)	.25
Mini-BESTest^f^ score, mean (SD)	19.83 (2.32)	22.17 (2.23)	.02	21.80 (3.42)		23.60 (3.13)	.31	20.50 (3.30)		23.00 (2.39)	.002
ABC^g^ score, mean (SD)	61.70 (24.22)	57.72 (21.41)	.66	83.44 (12.89)		70.79 (20.77)	.19	76.02 (14.82)		79.37 (11.36)	.42

^a^TR: telerehabilitation.

^b^PDQ: Parkinson’s Disease Questionnaire.

^c^TUG: timed up-and-go.

^d^STS: sit-to-stand.

^e^MWT: minute walk test.

^f^BESTest: balance evaluation systems test.

^g^ABC: Activities-Specific Balance Confidence Scale.

## Discussion

### Primary Findings

In this pilot randomized clinical trial of telerehabilitation for patients with PD, we screened 389 patient records for potential eligibility, included 20 patients after a series of exclusions, and analyzed data on satisfaction and clinical outcomes for 19 patients. In general, TR was found to be feasible and an accepted mode of therapy administration by both patients and therapists. Clinical outcomes were similar for patients in all groups, and patients and therapists were equally satisfied with the method for providing rehabilitation regardless of group allocation. However, our study uncovered some opportunities to consider optimal rehabilitation care pathways that could incorporate TR—or not—in patient-specific ways.

The primary reason for exclusion was the recent completion of a physical therapy episode of care, a criterion that was eliminated 2 years into the trial to encourage recruitment that had been significantly slowed by the COVID-19 pandemic. Many patients were also excluded due to an inability to schedule an initial appointment with the physical therapist for a research-specific visit (since the participating therapists maintained a usual clinical caseload throughout the duration of the study); this resulted in a low proportion of in-person screening of potentially eligible participants. In our stepwise method for applying exclusion criteria, very few patients were excluded for clinical reasons. Thus, if the exclusion criteria related to the timing of recent therapy episodes and ability to schedule can be appropriately managed in future trials, recruitment should be more feasible. None of the participants who enrolled voluntarily withdrew from the study, providing additional evidence of the feasibility of the study protocol.

Whether provided fully in-clinic (usual care), via TR only, or a hybrid including in-clinic visits with a TR-based home exercise program, patients and therapists generally agreed that the quantity and convenience of care delivery were adequate. Again, not differentiated by group, patients were generally satisfied with their home exercise program and its progression and felt safe during all components of the rehabilitation episode. Fewer patients in the TR groups believed that the prescribed exercises met their needs; the reason for this was not explored. One potential explanation is that the TR platform contained a finite library of PD-specific exercises from which therapists could choose. This may also explain why therapists rated their ability to provide and progress with an appropriate TR-based home exercise program lower than patients. Similarly, patients in the TR-only group generally rated their perception of safety higher than did the therapists for these same patients, and therapists were generally less likely than patients to recommend TR-only. While interesting, we did not conduct any statistical tests to compare patients’ responses to therapists’ responses since the sample size was too small and confounded by the fact that therapists responded multiple times (once for each patient for whom they provided care).

The survey responses (including the comments shown in Figure 2) highlight the potential for matching patients to the optimal utilization of a TR platform as part of the therapy episode. A few patients and therapists commented in some instances that a specific approach (ie, in-person, TR-only, or a hybrid model) would be preferred for certain patients. While our study design did not allow for cross-over outside of the allocated group, it would be reasonable to consider clinical models in which a patient’s presentation (eg, type and severity of deficits, distance lived from the clinic, comfort with technology, presence and ability of at-home caregivers, etc) could guide the use of TR. In such cases, shared decision-making would be an appropriate approach to planning [29,30].

A customized approach to the mode of rehabilitation delivery may be further justified by our findings that clinical outcomes were not different regardless of the care delivery approach. In this small sample, patients were not more or less likely to have differences in gait speed, balance, or self-reported function when using TR instead of or in conjunction with usual in-clinic visits. Others have reported similar results with TR in varied patient populations. A Cochrane review concluded that TR is equivalent to in-clinic rehabilitation for individuals with chronic respiratory disease [31]. Cottrell et al [32] found in a meta-analysis that TR alone leads to equivalent outcomes (function and pain) for patients with a primary musculoskeletal condition. They also concluded that TR as a supplement to in-clinic visits leads to better outcomes than either TR or in-clinic visits alone. In patients with neurological diseases, TR is feasible for patients with multiple sclerosis [33] and equivalent to traditional rehabilitation for patients with stroke [34]. Several researchers have highlighted the feasibility of conducting telehealth and general exercise prescription and monitoring for patients with PD [11,16,35-37] and a large trial is in process [38].

### Limitations

A few limitations should be noted. Since recruitment success was a primary outcome, protocol amendments that changed the eligibility criteria at 2 different points in the study influenced this outcome. In conjunction, these changes altered the characteristics of participants (12 who were continuing an active episode of therapy vs 7 who were starting a new episode) in ways that introduce bias that could not be reliably measured. Similarly, our sampling method was neither random nor consecutive since nearly 25% of patients who were clinically eligible at initial screening simply could not be scheduled for a visit due to a lack of therapist availability. Some individuals who met clinical criteria for participation did not have reliable internet and were therefore excluded. Future studies that provide Wi-Fi as needed would ensure that underserved populations are equally eligible and represented for TR interventions. Clinical outcomes were obtained by the study coordinator at baseline and the treating therapists post intervention, which may impact measurement reliability. Additionally, the therapist completing postintervention clinical testing was not blinded to group allocation, potentially introducing bias. Though intentional for this pilot trial, our small sample size (19 patients and 5 therapists) means that the results are preliminary and not generalizable to a larger population of similar patients with PD. These issues with recruitment and sampling need to be addressed in a larger trial.

### Conclusions

From this pilot randomized controlled trial of 20 patients at 2 rehabilitation centers, TR appears to be at least equivalent to usual rehabilitation in terms of patient and therapist satisfaction and clinical outcomes. An optimal care delivery strategy might include shared decision-making with patients so that TR is combined appropriately with in-person visits as part of the rehabilitation episode. This requires testing in future trials. In a future larger trial, eligibility criteria should be such that patients have similar recent experience with rehabilitation interventions, and the availability of therapists to provide the intervention needs to be assured so that a truly random sample can be recruited.

## Appendix

Multimedia Appendix 1 Patient and therapist satisfaction surveys.

Multimedia Appendix 2 Joint display of mixed quantitative and qualitative findings from satisfaction surveys.

Multimedia Appendix 3 CONSORT-EHEALTH (V 1.6.1) Submission Publication Form.

## Abbreviations

ABC: Activities-specific Balance Confidence Scale
CONSORT: Consolidated Standards of Reporting Trials
MDS-UPDRS III: Movement Disorder Society-sponsored Unified Parkinson’s Disease Rating Scale Part III
Mini-BESTest: mini balance evaluation systems test
MWT: minute walk test
PD: Parkinson disease
PDQ: Parkinson’s Disease Questionnaire
REDCap: Research Electronic Data Capture
TR: telerehabilitation
TUG: timed-up-and-go test
UC: usual care

## Data Availability

The data sets generated during and analyzed during this study are available from the corresponding author on reasonable request.
