# Supplementary material for: The Use of Telerehabilitation to Improve Movement-Related Outcomes and Quality of Life for Individuals With Parkinson Disease: Pilot Randomized Controlled Trial
Source: JMIR Form Res. 2024 Jul 31;8:e54599. doi: 10.2196/54599 (PMC11325111; doi:10.2196/54599)
Supplement: Multimedia Appendix 1 [file formative_v8i1e54599_app1.pdf]

Telerehabilitation for PD - Patient Satisfaction Survey

Please respond to each of the following in relation to your care in physical therapy over the past 12 weeks.

Thank you!

The following section asks about your "physical therapy treatment". This refers to the direct interactions that you had with the physical therapist over the past 12 weeks.

In which way did you primarily interact with your physical therapist?

◯ in-person visits ◯ video calls

Considering your [visit_type] with the physical therapist, rate the QUANTITY of these interactions.

◯ Very poor ◯ Poor ◯ Fair ◯ Good ◯ Very good

Would you say that you interacted with your physical therapist...

◯ Too much ◯ Too little

Considering your [visit_type] with the physical therapist, rate the CONVENIENCE of these interactions.

◯ Very poor ◯ Poor ◯ Fair ◯ Good ◯ Very good

Considering your [visit_type] with the physical therapist, rate the ability of your physical therapist to provide instructions to you regarding your exercises.

◯ Very poor ◯ Poor ◯ Fair ◯ Good ◯ Very good

The following section asks about your "home exercise program". This refers to the exercises that your physical therapist prescribed for you to complete on your own (unsupervised by the therapist) every day at home.

What was the primary way that you accessed your home exercise program?

◯ on paper ◯ online

Considering your home exercise program [hep_type], rate how well you believe these exercises met your needs.

○ Very poor ○ Poor ○ Fair ○ Good ○ Very good

Considering your home exercise program [hep_type], rate the ability of the physical therapist to provide you with an updated home exercise program.

○ Very poor ○ Poor ○ Fair ○ Good ○ Very good

Using the sliding scale below, please mark how safe you generally felt while completing your home exercise program?

Very unsafe Very safe

(Place a mark on the scale above)

On average, on how many days per week did you complete your home exercise program?

○ 1-2 days per week ○ 3-4 days per week ○ 5-7 days per week

The following section asks about your overall experience in physical therapy over the past 12 weeks. That is, the direct interaction you had with your physical therapist [visit_type] and the completion of your home exercise program, which was provided to you [hep_type].

Rate your OVERALL experience with completing your physical therapy treatment via [visit_type] and your home exercise program [hep_type].

◯ Very poor ◯ Poor ◯ Fair ◯ Good ◯ Very good

What made this a [patientrating_overall] experience for you?

How likely are you to recommend to another patient with Parkinson's disease that they complete their physical therapy treatment via [visit_type] and their home exercise program [hep_type]?

◯ Very unlikely ◯ Somewhat unlikely ◯ Unsure ◯ Somewhat likely ◯ Very likely

What additional comments do you have regarding your physical therapy treatment?

Telerehabilitation for PD - Provider Satisfaction Survey

Please respond to each of the following in relation to your care for this particular patient over the past 12 weeks.

Thank you!

The following section asks about "physical therapy treatment". This refers to the direct interaction you had with this patient over the past 12 weeks.

In which way did you primarily interact with this patient?

◯ in-person visits ◯ video calls

Considering your [prov_visit_type] with this patient, rate the QUANTITY of these interactions.

◯ Very poor ◯ Poor ◯ Fair ◯ Good ◯ Very good

Would you say that you interacted with this patient...

◯ Too much ◯ Too little

Considering your [prov_visit_type] with this patient, rate the CONVENIENCE of interacting with this patient.

◯ Very poor ◯ Poor ◯ Fair ◯ Good ◯ Very good

Considering your [prov_visit_type] with this patient, rate your ability to provide adequate instructions to the patient regarding his or her exercises.

◯ Very poor ◯ Poor ◯ Fair ◯ Good ◯ Very good

The following section asks about the "home exercise program". This refers to the exercises that you prescribed for this patient to complete on his or her own (unsupervised by you) at home.

What was the primary way that this patient accessed his or her home exercise program?

◯ on paper ◯ online

Considering this patient's home exercise program [prov_hep_type], rate how well you were able to provide exercises that met this patient's needs.

○ Very poor ○ Poor ○ Fair ○ Good ○ Very good

Considering this patient's home exercise program [prov_hep_type], rate your ability to make appropriate updates to the home exercise program.

○ Very poor ○ Poor ○ Fair ○ Good ○ Very good

Using the sliding scale below, please indicate how safe you feel the home exercise program was for this patient?

Very unsafe Very safe

(Place a mark on the scale above)

On average, on how many days do you believe that the patient completed his or her home exercise program?

○ 1-2 days per week ○ 3-4 days per week ○ 5-7 days per week

The following section asks about your overall experience in providing physical therapy treatment for this patient over the past 12 weeks. That is, the direct interaction you had with this patient via [prov_visit_type] and the completion of his or her home exercise program, which you provided to the patient [prov_hep_type].

Rate your OVERALL experience with providing physical therapy treatment [prov_visit_type] and the home exercise program [prov_hep_type].

◯ Very poor ◯ Poor ◯ Fair ◯ Good ◯ Very good

What made this a [provrating_overall] experience for you?

How likely are you in the future to combine physical therapy treatment via [prov_visit_type] and a home exercise program [prov_hep_type] for a similar patient with Parkinson's disease?

◯ Very unlikely ◯ Somewhat unlikely ◯ Unsure ◯ Somewhat likely ◯ Very likely

What additional comments do you have regarding this course of physical therapy treatment?
