# Supplementary material for: The Use of Telerehabilitation to Improve Movement-Related Outcomes and Quality of Life for Individuals With Parkinson Disease: Pilot Randomized Controlled Trial
Source: JMIR Form Res. 2024 Jul 31;8:e54599. doi: 10.2196/54599 (PMC11325111; doi:10.2196/54599)
Supplement: Multimedia Appendix 3 [file formative_v8i1e54599_app3.pdf]

CONSORT-EHEALTH (V 1.6.1) - Submission/Publication Form

The CONSORT-EHEALTH checklist is intended for authors of randomized trials evaluating web-based and Internet-based applications/interventions, including mobile interventions, electronic games (incl multiplayer games), social media, certain telehealth applications, and other interactive and/or networked electronic applications. Some of the items (e.g. all subitems under item 5 - description of the intervention) may also be applicable for other study designs.

The goal of the CONSORT EHEALTH checklist and guideline is to be
a) a guide for reporting for authors of RCTs,
b) to form a basis for appraisal of an ehealth trial (in terms of validity)

CONSORT-EHEALTH items/subitems are MANDATORY reporting items for studies published in the Journal of Medical Internet Research and other journals / scientific societies endorsing the checklist.

Items numbered 1., 2., 3., 4a., 4b etc are original CONSORT or CONSORT-NPT (non-pharmacologic treatment) items.
Items with Roman numerals (i., ii, iii, iv etc.) are CONSORT-EHEALTH extensions/clarifications.

As the CONSORT-EHEALTH checklist is still considered in a formative stage, we would ask that you also RATE ON A SCALE OF 1-5 how important/useful you feel each item is FOR THE PURPOSE OF THE CHECKLIST and reporting guideline (optional).

Mandatory reporting items are marked with a red *.
In the textboxes, either copy & paste the relevant sections from your manuscript into this form - please include any quotes from your manuscript in QUOTATION MARKS,
or answer directly by providing additional information not in the manuscript, or elaborating on why the item was not relevant for this study.

YOUR ANSWERS WILL BE PUBLISHED AS A SUPPLEMENTARY FILE TO YOUR PUBLICATION IN JMIR AND ARE CONSIDERED PART OF YOUR PUBLICATION (IF ACCEPTED).
Please fill in these questions diligently. Information will not be copyedited, so please use proper spelling and grammar, use correct capitalization, and avoid abbreviations.

DO NOT FORGET TO SAVE AS PDF _AND_ CLICK THE SUBMIT BUTTON SO YOUR ANSWERS ARE IN OUR DATABASE !!!

Citation Suggestion (if you append the pdf as Appendix we suggest to cite this paper in the caption):
Eysenbach G, CONSORT-EHEALTH Group

CONSORT-EHEALTH: Improving and Standardizing Evaluation Reports of Web-based and Mobile Health Interventions
J Med Internet Res 2011;13(4):e126
URL: http://www.jmir.org/2011/4/e126/
doi: 10.2196/jmir.1923
PMID: 22209829

johnson.j.2@gmail.com Switch account

Not shared

Draft saved

* Indicates required question

Your name *

First Last

Johnson

Primary Affiliation (short), City, Country *

University of Toronto, Toronto, Canada

Cleveland Clinic, Cleveland, United States

Your e-mail address *

abc@gmail.com

johnsoj8@ccf.org

Title of your manuscript *

Provide the (draft) title of your manuscript.

The use of telerehabilitation to improve movement-related outcomes and quality of life for individuals with Parkinson’s disease: A pilot randomized controlled trial

Name of your App/Software/Intervention *

If there is a short and a long/alternate name, write the short name first and add the long name in brackets.

WizeCare Ltd.

Evaluated Version (if any)

e.g. "V1", "Release 2017-03-01", "Version 2.0.27913"

Your answer

Language(s) *

What language is the intervention/app in? If multiple languages are available, separate by comma (e.g. "English, French")

English, Hebrew

URL of your Intervention Website or App

e.g. a direct link to the mobile app on app in appstore (itunes, Google Play), or URL of the website. If the intervention is a DVD or hardware, you can also link to an Amazon page.

https://www.wizecare.com/

URL of an image/screenshot (optional)

Your answer

Accessibility *

Can an enduser access the intervention presently?

- ○ access is free and open
- ○ access only for special usergroups, not open
- ◉ access is open to everyone, but requires payment/subscription/in-app purchases
- ○ app/intervention no longer accessible
- ○ Other:

Primary Medical Indication/Disease/Condition *

e.g. "Stress", "Diabetes", or define the target group in brackets after the condition, e.g. "Autism (Parents of children with)", "Alzheimers (Informal Caregivers of)"

Parkinson's disease

Primary Outcomes measured in trial *

comma-separated list of primary outcomes reported in the trial

Number eligible, number enrolled, proportion w

Secondary/other outcomes

Are there any other outcomes the intervention is expected to affect?

Timed-up-and-go test time, five-time sit-to-stand test time, 10-meter gait speed, six-minute walk test, mini balance evaluation systems test score, Parkinson's disease questionnaire, actitivities-specific balance confidence scale

Recommended "Dose" *

What do the instructions for users say on how often the app should be used?

- (x) Approximately Daily
- ( ) Approximately Weekly
- ( ) Approximately Monthly
- ( ) Approximately Yearly
- ( ) "as needed"
- ( ) Other:

Approx. Percentage of Users (starters) still using the app as recommended after 3 months *

- (x) unknown / not evaluated
- ( ) 0-10%
- ( ) 11-20%
- ( ) 21-30%
- ( ) 31-40%
- ( ) 41-50%
- ( ) 51-60%
- ( ) 61-70%
- ( ) 71%-80%
- ( ) 81-90%
- ( ) 91-100%
- ( ) Other:

Overall, was the app/intervention effective? *

- ○ yes: all primary outcomes were significantly better in intervention group vs control
- ○ partly: SOME primary outcomes were significantly better in intervention group vs control
- ○ no statistically significant difference between control and intervention
- ○ potentially harmful: control was significantly better than intervention in one or more outcomes
- ◉ inconclusive: more research is needed
- ○ Other:

Article Preparation Status/Stage *

At which stage in your article preparation are you currently (at the time you fill in this form)

- ○ not submitted yet - in early draft status
- ○ not submitted yet - in late draft status, just before submission
- ○ submitted to a journal but not reviewed yet
- ◉ submitted to a journal and after receiving initial reviewer comments
- ○ submitted to a journal and accepted, but not published yet
- ○ published
- ○ Other:

Journal *

If you already know where you will submit this paper (or if it is already submitted), please provide the journal name (if it is not JMIR, provide the journal name under "other")

- ○ not submitted yet / unclear where I will submit this
- ○ Journal of Medical Internet Research (JMIR)
- ○ JMIR mHealth and UHealth
- ○ JMIR Serious Games
- ○ JMIR Mental Health
- ○ JMIR Public Health
- ◉ JMIR Formative Research
- ○ Other JMIR sister journal
- ○ Other:

Is this a full powered effectiveness trial or a pilot/feasibility trial? *

- ◉ Pilot/feasibility
- ○ Fully powered

Manuscript tracking number *

If this is a JMIR submission, please provide the manuscript tracking number under "other" (The ms tracking number can be found in the submission acknowledgement email, or when you login as author in JMIR. If the paper is already published in JMIR, then the ms tracking number is the four-digit number at the end of the DOI, to be found at the bottom of each published article in JMIR)

- ○ no ms number (yet) / not (yet) submitted to / published in JMIR
- ◉ Other: JFR ms#54599

TITLE AND ABSTRACT

1a) TITLE: Identification as a randomized trial in the title

1a) Does your paper address CONSORT item 1a? *

I.e does the title contain the phrase "Randomized Controlled Trial"? (if not, explain the reason under "other")

- (x) yes
- ( ) Other:

1a-i) Identify the mode of delivery in the title

Identify the mode of delivery. Preferably use “web-based” and/or “mobile” and/or “electronic game” in the title. Avoid ambiguous terms like “online”, “virtual”, “interactive”. Use “Internet-based” only if Intervention includes non-web-based Internet components (e.g. email), use “computer-based” or “electronic” only if offline products are used. Use “virtual” only in the context of “virtual reality” (3-D worlds). Use “online” only in the context of “online support groups”. Complement or substitute product names with broader terms for the class of products (such as “mobile” or “smart phone” instead of “iphone”), especially if the application runs on different platforms.

	1	2	3	4	5	
subitem not at all important	( )	( )	(x)	( )	( )	essential

Clear selection

Does your paper address subitem 1a-i? *

Copy and paste relevant sections from manuscript title (include quotes in quotation marks "like this" to indicate direct quotes from your manuscript), or elaborate on this item by providing additional information not in the ms, or briefly explain why the item is not applicable/relevant for your study

Patients and providers accessed this telerehabilitation platform using both a mobile application and the internet, since both are available. This is difficult to describe in a brief title.

1a-ii) Non-web-based components or important co-interventions in title

Mention non-web-based components or important co-interventions in title, if any (e.g., “with telephone support”).

	1	2	3	4	5	
subitem not at all important	○	●	○	○	○	essential

Clear selection

Does your paper address subitem 1a-ii?

Copy and paste relevant sections from manuscript title (include quotes in quotation marks "like this" to indicate direct quotes from your manuscript), or elaborate on this item by providing additional information not in the ms, or briefly explain why the item is not applicable/relevant for your study

The non-web-based components were used for the standard-of-care and hybrid comparison groups, which is difficult to describe in a brief title.

1a-iii) Primary condition or target group in the title

Mention primary condition or target group in the title, if any (e.g., “for children with Type I Diabetes”) Example: A Web-based and Mobile Intervention with Telephone Support for Children with Type I Diabetes: Randomized Controlled Trial

	1	2	3	4	5	
subitem not at all important	○	○	○	○	◉	essential

Clear selection

Does your paper address subitem 1a-iii? *

Copy and paste relevant sections from manuscript title (include quotes in quotation marks "like this" to indicate direct quotes from your manuscript), or elaborate on this item by providing additional information not in the ms, or briefly explain why the item is not applicable/relevant for your study

"individuals with Parkinson’s disease"

1b) ABSTRACT: Structured summary of trial design, methods, results, and conclusions

NPT extension: Description of experimental treatment, comparator, care providers, centers, and blinding status.

1b-i) Key features/functionalities/components of the intervention and comparator in the METHODS section of the ABSTRACT

Mention key features/functionalities/components of the intervention and comparator in the abstract. If possible, also mention theories and principles used for designing the site. Keep in mind the needs of systematic reviewers and indexers by including important synonyms. (Note: Only report in the abstract what the main paper is reporting. If this information is missing from the main body of text, consider adding it)

	1	2	3	4	5	
subitem not at all important	○	○	○	◉	○	essential

Clear selection

Does your paper address subitem 1b-i? *

Copy and paste relevant sections from the manuscript abstract (include quotes in quotation marks "like this" to indicate direct quotes from your manuscript), or elaborate on this item by providing additional information not in the ms, or briefly explain why the item is not applicable/relevant for your study

"TR was administered using a web-based application with a mobile application option. One-hour interventions were performed weekly for four weeks, in-person for the clinic+TR and UC groups and virtually for the TR-only group. Home exercises were provided on paper for the UC group and via the virtual platform for the clinic+TR and TR-only groups."

1b-ii) Level of human involvement in the METHODS section of the ABSTRACT

Clarify the level of human involvement in the abstract, e.g., use phrases like “fully automated” vs. “therapist/nurse/care provider/physician-assisted” (mention number and expertise of providers involved, if any). (Note: Only report in the abstract what the main paper is reporting. If this information is missing from the main body of text, consider adding it)

	1	2	3	4	5	
subitem not at all important	◉	○	○	○	○	essential

Clear selection

Does your paper address subitem 1b-ii?

Copy and paste relevant sections from the manuscript abstract (include quotes in quotation marks "like this" to indicate direct quotes from your manuscript), or elaborate on this item by providing additional information not in the ms, or briefly explain why the item is not applicable/relevant for your study

This is described with greater detail in the body of the manuscript.

1b-iii) Open vs. closed, web-based (self-assessment) vs. face-to-face assessments in the METHODS section of the ABSTRACT

Mention how participants were recruited (online vs. offline), e.g., from an open access website or from a clinic or a closed online user group (closed usergroup trial), and clarify if this was a purely web-based trial, or there were face-to-face components (as part of the intervention or for assessment). Clearly say if outcomes were self-assessed through questionnaires (as common in web-based trials). Note: In traditional offline trials, an open trial (open-label trial) is a type of clinical trial in which both the researchers and participants know which treatment is being administered. To avoid confusion, use “blinded” or “unblinded” to indicated the level of blinding instead of “open”, as “open” in web-based trials usually refers to “open access” (i.e. participants can self-enrol). (Note: Only report in the abstract what the main paper is reporting. If this information is missing from the main body of text, consider adding it)

	1	2	3	4	5	
subitem not at all important	○	○	◉	○	○	essential

Clear selection

Does your paper address subitem 1b-iii?

Copy and paste relevant sections from the manuscript abstract (include quotes in quotation marks "like this" to indicate direct quotes from your manuscript), or elaborate on this item by providing additional information not in the ms, or briefly explain why the item is not applicable/relevant for your study

"This was a pilot randomized controlled trial conducted at two outpatient neurorehabilitation clinics"

1b-iv) RESULTS section in abstract must contain use data

Report number of participants enrolled/assessed in each group, the use/uptake of the intervention (e.g., attrition/adherence metrics, use over time, number of logins etc.), in addition to primary/secondary outcomes. (Note: Only report in the abstract what the main paper is reporting. If this information is missing from the main body of text, consider adding it)

	1	2	3	4	5	
subitem not at all important	○	○	○	○	◉	essential

Clear selection

Does your paper address subitem 1b-iv?

Copy and paste relevant sections from the manuscript abstract (include quotes in quotation marks "like this" to indicate direct quotes from your manuscript), or elaborate on this item by providing additional information not in the ms, or briefly explain why the item is not applicable/relevant for your study

"Of 389 patients screened, 68 (17.5%) met eligibility criteria and 20 (29.4% of those eligible) were enrolled (Clinic+TR, N=6; TR-only, N=6; usual care, N=8). One patient (TR-only) was withdrawn due to a non-study related injurious fall. "

1b-v) CONCLUSIONS/DISCUSSION in abstract for negative trials

Conclusions/Discussions in abstract for negative trials: Discuss the primary outcome - if the trial is negative (primary outcome not changed), and the intervention was not used, discuss whether negative results are attributable to lack of uptake and discuss reasons. (Note: Only report in the abstract what the main paper is reporting. If this information is missing from the main body of text, consider adding it)

	1	2	3	4	5	
subitem not at all important	◉	○	○	○	○	essential

Clear selection

Does your paper address subitem 1b-v?

Copy and paste relevant sections from the manuscript abstract (include quotes in quotation marks "like this" to indicate direct quotes from your manuscript), or elaborate on this item by providing additional information not in the ms, or briefly explain why the item is not applicable/relevant for your study

As a feasibility trial, the primary outcomes were descriptive, related to trial enrollment and retention.

INTRODUCTION

2a) In INTRODUCTION: Scientific background and explanation of rationale

2a-i) Problem and the type of system/solution

Describe the problem and the type of system/solution that is object of the study: intended as stand-alone intervention vs. incorporated in broader health care program? Intended for a particular patient population? Goals of the intervention, e.g., being more cost-effective to other interventions, replace or complement other solutions? (Note: Details about the intervention are provided in “Methods” under 5)

	1	2	3	4	5	
subitem not at all important	○	○	○	○	◉	essential

Clear selection

Does your paper address subitem 2a-i? *

Copy and paste relevant sections from the manuscript (include quotes in quotation marks "like this" to indicate direct quotes from your manuscript), or elaborate on this item by providing additional information not in the ms, or briefly explain why the item is not applicable/relevant for your study

"Individuals with Parkinson’s disease (PD) can improve overall mobility and participation in daily activities as they engage in frequent exercise.[1], [2], [3], [4], [5], [6] Further, exercise may provide a neuroprotective effect for these individuals, thereby limiting the progression of the disease.[7] As in other patient populations, individuals with PD demonstrate the greatest benefit from an exercise program that is designed to meet their specific needs.[8], [9]
Despite the need for individually tailored exercise programs, persons with PD often face barriers to accessing physical rehabilitation professionals who can provide them. Patients may live too far from a specialized clinic, be unable to secure adequate transportation to a clinic, or face structural barriers if the clinic’s physical environment is not conducive to allowing safe movement throughout.[10], [11] Additionally, system-level barriers like limitations in the number of adequately trained rehabilitation clinicians and poorly aligned reimbursement policies may limit the frequency with which persons with PD can receive the specified exercise interventions that may be of greatest benefit to them.[12], [13]"

2a-ii) Scientific background, rationale: What is known about the (type of) system

Scientific background, rationale: What is known about the (type of) system that is the object of the study (be sure to discuss the use of similar systems for other conditions/diagnoses, if appropiate), motivation for the study, i.e. what are the reasons for and what is the context for this specific study, from which stakeholder viewpoint is the study performed, potential impact of findings [2]. Briefly justify the choice of the comparator.

	1	2	3	4	5	
subitem not at all important	○	○	○	●	○	essential

Clear selection

Does your paper address subitem 2a-ii? *

Copy and paste relevant sections from the manuscript (include quotes in quotation marks "like this" to indicate direct quotes from your manuscript), or elaborate on this item by providing additional information not in the ms, or briefly explain why the item is not applicable/relevant for your study

"Medical care—“telemedicine”—via phone or video calls is preferred by many patients with PD and their often over-burdened caregivers as it is more convenient and less costly.[11] Its extension to rehabilitation—"telerehabilitation”—can address many of the barriers faced by those with PD to receive specialized care.[14], [15] Prescribing at-home exercises is a common practice of physical therapists; however, generic home exercise programs that do not incorporate disease- and patient-specific components are not beneficial.[8] Customized programs that incorporate PD-specific exercises that are individually tailored to address an individual’s symptoms and goals, however, may be feasible and effective.[16], [17]"

2b) In INTRODUCTION: Specific objectives or hypotheses

Does your paper address CONSORT subitem 2b? *

Copy and paste relevant sections from the manuscript (include quotes in quotation marks "like this" to indicate direct quotes from your manuscript), or elaborate on this item by providing additional information not in the ms, or briefly explain why the item is not applicable/relevant for your study

"The primary aim of this pilot randomized controlled trial was to assess the feasibility of telerehabilitation (TR) for patients with PD by examining recruitment and retention success and exploring perceptions of satisfaction among patients and the physical therapists that provided their interventions. We also conducted a preliminary exploratory examination of the clinical effectiveness of TR for persons with PD."

METHODS

3a) Description of trial design (such as parallel, factorial) including allocation ratio

Does your paper address CONSORT subitem 3a? *

Copy and paste relevant sections from the manuscript (include quotes in quotation marks "like this" to indicate direct quotes from your manuscript), or elaborate on this item by providing additional information not in the ms, or briefly explain why the item is not applicable/relevant for your study

"This was an unblinded randomized controlled trial."

3b) Important changes to methods after trial commencement (such as eligibility criteria), with reasons

Does your paper address CONSORT subitem 3b? *

Copy and paste relevant sections from the manuscript (include quotes in quotation marks "like this" to indicate direct quotes from your manuscript), or elaborate on this item by providing additional information not in the ms, or briefly explain why the item is not applicable/relevant for your study

"Though not an a priori criterion, some patients could not be scheduled for study participation due to a lack of availability on the schedules of participating therapists. "

"The protocol was amended significantly twice for the sake of encouraging enrollment, which was slowed due to the COVID-19 pandemic and slow return of patients with PD to outpatient rehabilitation clinics. The first amendment (in November 2020) changed the criterion for the timing of any recent outpatient physical therapy episode from 5 months to 6 weeks. The second amendment (April 2022) eliminated the criterion related to recent outpatient physical therapy episodes meaning that patients could be identified for pre-screening even while participating in an ongoing physical therapy care episode."

3b-i) Bug fixes, Downtimes, Content Changes

Bug fixes, Downtimes, Content Changes: ehealth systems are often dynamic systems. A description of changes to methods therefore also includes important changes made on the intervention or comparator during the trial (e.g., major bug fixes or changes in the functionality or content) (5-iii) and other “unexpected events” that may have influenced study design such as staff changes, system failures/downtimes, etc. [2].

	1	2	3	4	5	
subitem not at all important	(•)	( )	( )	( )	( )	essential

Clear selection

Does your paper address subitem 3b-i?

Copy and paste relevant sections from the manuscript (include quotes in quotation marks "like this" to indicate direct quotes from your manuscript), or elaborate on this item by providing additional information not in the ms, or briefly explain why the item is not applicable/relevant for your study

This did not occur during the conduct of this trial.

4a) Eligibility criteria for participants

Does your paper address CONSORT subitem 4a? *

Copy and paste relevant sections from the manuscript (include quotes in quotation marks "like this" to indicate direct quotes from your manuscript), or elaborate on this item by providing additional information not in the ms, or briefly explain why the item is not applicable/relevant for your study

"Patients were recruited between February 1, 2020 and December 15, 2022. Initially, patients were identified for pre-screening if they had a diagnosis of PD for any duration and were scheduled for a new episode of physical therapy. Pre-screening included chart review by a study coordinator. The exclusion criteria were: any outpatient physical therapy episode in the preceding 5 months, documented diagnosis of another progressive neurological disease, history of epilepsy, moderate to severe cognitive impairment, hemiparesis or hemiplegia, current complaints of dizziness."

"At pre-screen phone call, patients were excluded if they were not reached, reported not having a reliable caregiver or internet connection in their home, or declined to participate."

"Final eligibility was determined by completion of the timed-up-and-go test (TUG) in <15 seconds, a 10-meter gait speed of >0.8 m/s, and a subjective determination of safety by the evaluating physical therapist."

4a-i) Computer / Internet literacy

Computer / Internet literacy is often an implicit “de facto” eligibility criterion - this should be explicitly clarified.

	1	2	3	4	5	
subitem not at all important	○	○	◉	○	○	essential

Clear selection

Does your paper address subitem 4a-i?

Copy and paste relevant sections from the manuscript (include quotes in quotation marks "like this" to indicate direct quotes from your manuscript), or elaborate on this item by providing additional information not in the ms, or briefly explain why the item is not applicable/relevant for your study

"At pre-screen phone call, patients were excluded if they were not reached, reported not having a reliable caregiver or internet connection in their home, or declined to participate."

4a-ii) Open vs. closed, web-based vs. face-to-face assessments:

Open vs. closed, web-based vs. face-to-face assessments: Mention how participants were recruited (online vs. offline), e.g., from an open access website or from a clinic, and clarify if this was a purely web-based trial, or there were face-to-face components (as part of the intervention or for assessment), i.e., to what degree got the study team to know the participant. In online-only trials, clarify if participants were quasi-anonymous and whether having multiple identities was possible or whether technical or logistical measures (e.g., cookies, email confirmation, phone calls) were used to detect/prevent these.

	1	2	3	4	5	
subitem not at all important	○	○	○	◉	○	essential

Clear selection

Does your paper address subitem 4a-ii? *

Copy and paste relevant sections from the manuscript (include quotes in quotation marks "like this" to indicate direct quotes from your manuscript), or elaborate on this item by providing additional information not in the ms, or briefly explain why the item is not applicable/relevant for your study

"After chart review and phone screening, those interested in participation completed the informed consent process in-person administered by a study coordinator and further screened for eligibility by the evaluating physical therapist immediately prior to their initial physical therapy visit."

4a-iii) Information giving during recruitment

Information given during recruitment. Specify how participants were briefed for recruitment and in the informed consent procedures (e.g., publish the informed consent documentation as appendix, see also item X26), as this information may have an effect on user self-selection, user expectation and may also bias results.

	1	2	3	4	5	
subitem not at all important	○	○	○	●	○	essential

Clear selection

Does your paper address subitem 4a-iii?

Copy and paste relevant sections from the manuscript (include quotes in quotation marks "like this" to indicate direct quotes from your manuscript), or elaborate on this item by providing additional information not in the ms, or briefly explain why the item is not applicable/relevant for your study

"After chart review and phone screening, those interested in participation completed the informed consent process in-person administered by a study coordinator and further screened for eligibility by the evaluating physical therapist immediately prior to their initial physical therapy visit."

4b) Settings and locations where the data were collected

Does your paper address CONSORT subitem 4b? *

Copy and paste relevant sections from the manuscript (include quotes in quotation marks "like this" to indicate direct quotes from your manuscript), or elaborate on this item by providing additional information not in the ms, or briefly explain why the item is not applicable/relevant for your study

"The study was conducted at two Cleveland Clinic outpatient rehabilitation clinics in Cleveland, Ohio, USA and Las Vegas, Nevada, USA. Both outpatient clinics specialize in rehabilitation for degenerative neurological diseases."

4b-i) Report if outcomes were (self-)assessed through online questionnaires

Clearly report if outcomes were (self-)assessed through online questionnaires (as common in web-based trials) or otherwise.

	1	2	3	4	5	
subitem not at all important	○	○	○	◉	○	essential

Clear selection

Does your paper address subitem 4b-i? *

Copy and paste relevant sections from the manuscript (include quotes in quotation marks "like this" to indicate direct quotes from your manuscript), or elaborate on this item by providing additional information not in the ms, or briefly explain why the item is not applicable/relevant for your study

"For all groups, we assessed satisfaction with the mode of care delivery using surveys, which were administered to the patient and the physical therapist at the in-clinic discharge visit via the Research Electronic Data Capture (REDCap) platform."

4b-ii) Report how institutional affiliations are displayed

Report how institutional affiliations are displayed to potential participants [on ehealth media], as affiliations with prestigious hospitals or universities may affect volunteer rates, use, and reactions with regards to an intervention.(Not a required item – describe only if this may bias results)

	1	2	3	4	5	
subitem not at all important	○	○	○	○	○	essential

Does your paper address subitem 4b-ii?

Copy and paste relevant sections from the manuscript (include quotes in quotation marks "like this" to indicate direct quotes from your manuscript), or elaborate on this item by providing additional information not in the ms, or briefly explain why the item is not applicable/relevant for your study

Your answer

5) The interventions for each group with sufficient details to allow replication, including how and when they were actually administered

5-i) Mention names, credential, affiliations of the developers, sponsors, and owners

Mention names, credential, affiliations of the developers, sponsors, and owners [6] (if authors/evaluators are owners or developer of the software, this needs to be declared in a "Conflict of interest" section or mentioned elsewhere in the manuscript).

	1	2	3	4	5	
subitem not at all important	○	○	○	●	○	essential

Clear selection

Does your paper address subitem 5-i?

Copy and paste relevant sections from the manuscript (include quotes in quotation marks "like this" to indicate direct quotes from your manuscript), or elaborate on this item by providing additional information not in the ms, or briefly explain why the item is not applicable/relevant for your study

"WizeCare Technologies (Or Yehuda, Israel) funded this study. The sponsor provided mobile tablets as an option for patient use. Otherwise, WizeCare played no role in the design, execution, or reporting of the study."

5-ii) Describe the history/development process

Describe the history/development process of the application and previous formative evaluations (e.g., focus groups, usability testing), as these will have an impact on adoption/use rates and help with interpreting results.

	1	2	3	4	5	
subitem not at all important	○	○	●	○	○	essential

Clear selection

Does your paper address subitem 5-ii?

Copy and paste relevant sections from the manuscript (include quotes in quotation marks "like this" to indicate direct quotes from your manuscript), or elaborate on this item by providing additional information not in the ms, or briefly explain why the item is not applicable/relevant for your study

"The WizeCare TR platform enables remote rehabilitation training and monitoring using a customized combination of video conferencing and libraries of pre-recorded videos of specific exercises. The videos were combined manually to create customized exercise prescriptions for patients that varied in content and duration. Immediately prior to the study, a series of videos of common PD-specific exercises were produced and stored in a specific library on the platform."

5-iii) Revisions and updating

Revisions and updating. Clearly mention the date and/or version number of the application/intervention (and comparator, if applicable) evaluated, or describe whether the intervention underwent major changes during the evaluation process, or whether the development and/or content was “frozen” during the trial. Describe dynamic components such as news feeds or changing content which may have an impact on the replicability of the intervention (for unexpected events see item 3b).

	1	2	3	4	5	
subitem not at all important	○	○	●	○	○	essential

Clear selection

Does your paper address subitem 5-iii?

Copy and paste relevant sections from the manuscript (include quotes in quotation marks "like this" to indicate direct quotes from your manuscript), or elaborate on this item by providing additional information not in the ms, or briefly explain why the item is not applicable/relevant for your study

"No updates were incorporated to the TR platform during the conduct of this trial."

5-iv) Quality assurance methods

Provide information on quality assurance methods to ensure accuracy and quality of information provided [1], if applicable.

	1	2	3	4	5	
subitem not at all important	○	◉	○	○	○	essential

Clear selection

Does your paper address subitem 5-iv?

Copy and paste relevant sections from the manuscript (include quotes in quotation marks "like this" to indicate direct quotes from your manuscript), or elaborate on this item by providing additional information not in the ms, or briefly explain why the item is not applicable/relevant for your study

"Additionally, the WizeCare platform enabled a safety assessment with each session in which a patient interacted with the system, including their unsupervised home exercise sessions. At the conclusion of each session on the platform, patients were asked, “Did you feel safe during this session?” If they answered “No”, the system sent an automated message to the treating physical therapist. The system also provided an automated response to the patient to, “Please stop participating in the exercise program and call your treating physical therapist.” Initiated by either the physical therapist or the patient within 24 hours of the reported lack of safety, a conversation between them allowed the patient to express any safety concerns. Any such events were recorded as adverse events, with subsequent steps as outlined above. These patient self-reports of safety were included amongst exploratory outcomes."

5-v) Ensure replicability by publishing the source code, and/or providing screenshots/screen-capture video, and/or providing flowcharts of the algorithms used

Ensure replicability by publishing the source code, and/or providing screenshots/screen-capture video, and/or providing flowcharts of the algorithms used. Replicability (i.e., other researchers should in principle be able to replicate the study) is a hallmark of scientific reporting.

	1	2	3	4	5	
subitem not at all important	○	◉	○	○	○	essential

Clear selection

Does your paper address subitem 5-v?

Copy and paste relevant sections from the manuscript (include quotes in quotation marks "like this" to indicate direct quotes from your manuscript), or elaborate on this item by providing additional information not in the ms, or briefly explain why the item is not applicable/relevant for your study

Per the journal guidelines, the following statement is included: "The data sets generated during and/or analyzed during this study are available from the corresponding author on reasonable request."

5-vi) Digital preservation

Digital preservation: Provide the URL of the application, but as the intervention is likely to change or disappear over the course of the years; also make sure the intervention is archived (Internet Archive, webcitation.org, and/or publishing the source code or screenshots/videos alongside the article). As pages behind login screens cannot be archived, consider creating demo pages which are accessible without login.

	1	2	3	4	5	
subitem not at all important	◉	○	○	○	○	essential

Clear selection

Does your paper address subitem 5-vi?

Copy and paste relevant sections from the manuscript (include quotes in quotation marks "like this" to indicate direct quotes from your manuscript), or elaborate on this item by providing additional information not in the ms, or briefly explain why the item is not applicable/relevant for your study

We can provide a direct link to the online WizeCare platform if requested by the journal to do so.

5-vii) Access

Access: Describe how participants accessed the application, in what setting/context, if they had to pay (or were paid) or not, whether they had to be a member of specific group. If known, describe how participants obtained “access to the platform and Internet” [1]. To ensure access for editors/reviewers/readers, consider to provide a “backdoor” login account or demo mode for reviewers/readers to explore the application (also important for archiving purposes, see vi).

	1	2	3	4	5	
subitem not at all important	○	○	○	◉	○	essential

Clear selection

Does your paper address subitem 5-vii? *

Copy and paste relevant sections from the manuscript (include quotes in quotation marks "like this" to indicate direct quotes from your manuscript), or elaborate on this item by providing additional information not in the ms, or briefly explain why the item is not applicable/relevant for your study

"Patients completed TR on a tablet (Lenovo Yoga Tab 3, Model YT3-X50F, OS Android 6.0) provided by WizeCare, their own tablet, their own laptop or desktop computer, or their own cell phone. If by tablet or cell phone, the platform was accessed using a mobile app. If by computer, the platform was accessed via a web browser."

"Volunteer participants were not compensated for their participation, a point that was described during the informed consent process."

5-viii) Mode of delivery, features/functionalities/components of the intervention and comparator, and the theoretical framework

Describe mode of delivery, features/functionalities/components of the intervention and comparator, and the theoretical framework [6] used to design them (instructional strategy [1], behaviour change techniques, persuasive features, etc., see e.g., [7, 8] for terminology). This includes an in-depth description of the content (including where it is coming from and who developed it) [1],” whether [and how] it is tailored to individual circumstances and allows users to track their progress and receive feedback” [6]. This also includes a description of communication delivery channels and – if computer-mediated communication is a component – whether communication was synchronous or asynchronous [6]. It also includes information on presentation strategies [1], including page design principles, average amount of text on pages, presence of hyperlinks to other resources, etc. [1].

	1	2	3	4	5	
subitem not at all important	○	○	○	●	○	essential

Clear selection

Does your paper address subitem 5-viii? *

Copy and paste relevant sections from the manuscript (include quotes in quotation marks "like this" to indicate direct quotes from your manuscript), or elaborate on this item by providing additional information not in the ms, or briefly explain why the item is not applicable/relevant for your study

"The WizeCare TR platform enables remote rehabilitation training and monitoring using a customized combination of video conferencing and libraries of pre-recorded videos of specific exercises. The videos were combined manually to create customized exercise prescriptions for patients that varied in content and duration. Immediately prior to the study, a series of videos of common PD-specific exercises were produced and stored in a specific library on the platform. Physical therapists built customized and individualized home exercise programs primarily from this library, though videos from any library were available for use. The platform also featured an option for live video calls between patients and therapists, which enabled ongoing communication and re-evaluation for adjustment of the rehabilitation plan as needed."

5-ix) Describe use parameters

Describe use parameters (e.g., intended "doses" and optimal timing for use). Clarify what instructions or recommendations were given to the user, e.g., regarding timing, frequency, heaviness of use, if any, or was the intervention used ad libitum.

	1	2	3	4	5	
subitem not at all important	○	○	○	◉	○	essential

Clear selection

Does your paper address subitem 5-ix?

Copy and paste relevant sections from the manuscript (include quotes in quotation marks "like this" to indicate direct quotes from your manuscript), or elaborate on this item by providing additional information not in the ms, or briefly explain why the item is not applicable/relevant for your study

"Each participant was in the study for five weeks, which included an initial evaluation, four weeks of therapy, and a discharge visit. For all four therapy weeks, those in the clinic+TR group participated in physical therapy once per week in the clinic and in a daily therapist-prescribed home exercise program using the TR platform. Those in the TR-only group also participated in a daily therapist-prescribed home exercise program using the platform, but with once weekly virtual visits (via video conference calls) taking the place of clinic visits. Those in the usual care group participated in physical therapy once per week in the clinic and in a daily therapist-prescribed home exercise program provided on a printed paper handout."

5-x) Clarify the level of human involvement

Clarify the level of human involvement (care providers or health professionals, also technical assistance) in the e-intervention or as co-intervention (detail number and expertise of professionals involved, if any, as well as "type of assistance offered, the timing and frequency of the support, how it is initiated, and the medium by which the assistance is delivered". It may be necessary to distinguish between the level of human involvement required for the trial, and the level of human involvement required for a routine application outside of a RCT setting (discuss under item 21 – generalizability).

	1	2	3	4	5	
subitem not at all important	○	○	◉	○	○	essential

Clear selection

Does your paper address subitem 5-x?

Copy and paste relevant sections from the manuscript (include quotes in quotation marks "like this" to indicate direct quotes from your manuscript), or elaborate on this item by providing additional information not in the ms, or briefly explain why the item is not applicable/relevant for your study

"Each participant was in the study for five weeks, which included an initial evaluation, four weeks of therapy, and a discharge visit. For all four therapy weeks, those in the clinic+TR group participated in physical therapy once per week in the clinic and in a daily therapist-prescribed home exercise program using the TR platform. Those in the TR-only group also participated in a daily therapist-prescribed home exercise program using the platform, but with once weekly virtual visits (via video conference calls) taking the place of clinic visits. Those in the usual care group participated in physical therapy once per week in the clinic and in a daily therapist-prescribed home exercise program provided on a printed paper handout."

"The WizeCare TR platform enables remote rehabilitation training and monitoring using a customized combination of video conferencing and libraries of pre-recorded videos of specific exercises. The videos were combined manually to create customized exercise prescriptions for patients that varied in content and duration. Immediately prior to the study, a series of videos of common PD-specific exercises were produced and stored in a specific library on the platform. Physical therapists built customized and individualized home exercise programs primarily from this library, though videos from any library were available for use. The platform also featured an option for live video calls between patients and therapists, which enabled ongoing communication and re-evaluation for adjustment of the rehabilitation plan as needed."

5-xi) Report any prompts/reminders used

Report any prompts/reminders used: Clarify if there were prompts (letters, emails, phone calls, SMS) to use the application, what triggered them, frequency etc. It may be necessary to distinguish between the level of prompts/reminders required for the trial, and the level of prompts/reminders for a routine application outside of a RCT setting (discuss under item 21 – generalizability).

	1	2	3	4	5	
subitem not at all important	(selected)					essential

Clear selection

Does your paper address subitem 5-xi? *

Copy and paste relevant sections from the manuscript (include quotes in quotation marks "like this" to indicate direct quotes from your manuscript), or elaborate on this item by providing additional information not in the ms, or briefly explain why the item is not applicable/relevant for your study

We did not use prompts/reminders since visits were scheduled.

5-xii) Describe any co-interventions (incl. training/support)

Describe any co-interventions (incl. training/support): Clearly state any interventions that are provided in addition to the targeted eHealth intervention, as ehealth intervention may not be designed as stand-alone intervention. This includes training sessions and support [1]. It may be necessary to distinguish between the level of training required for the trial, and the level of training for a routine application outside of a RCT setting (discuss under item 21 – generalizability.

	1	2	3	4	5	
subitem not at all important	(selected)					essential

Clear selection

Does your paper address subitem 5-xii? *

Copy and paste relevant sections from the manuscript (include quotes in quotation marks "like this" to indicate direct quotes from your manuscript), or elaborate on this item by providing additional information not in the ms, or briefly explain why the item is not applicable/relevant for your study

There were no co-interventions.

6a) Completely defined pre-specified primary and secondary outcome measures, including how and when they were assessed

Does your paper address CONSORT subitem 6a? *

Copy and paste relevant sections from the manuscript (include quotes in quotation marks "like this" to indicate direct quotes from your manuscript), or elaborate on this item by providing additional information not in the ms, or briefly explain why the item is not applicable/relevant for your study

"As a pilot study, the primary outcomes of interest were recruitment success (number eligible and number enrolled), the proportion who withdrew from the study for any reason, and satisfaction amongst patients and therapists. For all groups, we assessed satisfaction with the mode of care delivery using surveys, which were administered to the patient and the physical therapist at the in-clinic discharge visit via the Research Electronic Data Capture (REDCap) platform.[18] Participating physical therapists responded after each care episode, considering the mode of delivery for that specific patient. Each survey assessed eight constructs; six satisfaction items were rated on a 5-point Likert scale (“very poor”, “poor”, “fair”, “good”, or “very good”), one item pertaining to safety used a 0 (“very unsafe”) to 100 (“very safe”) rating, and one item asked how likely the respondent would be to recommend their mode of delivery (for patient respondents) or use the same mode of delivery for a future similar patient (for therapist respondents). A final open-ended question sought any other written feedback. The surveys were designed by the principal investigator then pilot tested by non-clinical members of the study team (since the clinical members of the study team would be completing the surveys as part of the study). The survey questions are presented in Appendix 1.
Clinical measures were assessed at baseline by a study coordinator and at five weeks by the treating therapist using instruments that are reliable, valid, and sensitive to change in patients with PD. These included the timed-up-and-go (TUG) test,[21], [22] TUG cognitive task assessment,[23] the five-time sit-to-stand (5STS) test,[24], [25] the 10-meter walk test, [21] the six-minute walk test (6MWT),[26] and the Mini Balance Evaluation Systems Test (Mini-BESTest).[27] Additionally, patients’ self-assessment of function was collected using the Parkinson’s Disease Questionnaire (PDQ-39)[28] and the Activities-specific Balance Confidence Scale (ABC),[22] both of which have been validated for use by patients with PD. The PDQ-39 summary index was used, which scales scores to 0-100 with higher scores indicating greater difficulty with tasks. Higher ABC scores indicate greater confidence."

6a-i) Online questionnaires: describe if they were validated for online use and apply CHERRIES items to describe how the questionnaires were designed/deployed

If outcomes were obtained through online questionnaires, describe if they were validated for online use and apply CHERRIES items to describe how the questionnaires were designed/deployed [9].

	1	2	3	4	5	
subitem not at all important	(•)	○	○	○	○	essential

Clear selection

Does your paper address subitem 6a-i?

Copy and paste relevant sections from manuscript text

Surveys were administered in REDCap during in-person visits at baseline and discharge.

6a-ii) Describe whether and how "use" (including intensity of use/dosage) was defined/measured/monitored

Describe whether and how "use" (including intensity of use/dosage) was defined/measured/monitored (logins, logfile analysis, etc.). Use/adoption metrics are important process outcomes that should be reported in any ehealth trial.

	1	2	3	4	5	
subitem not at all important	○	○	○	○	○	essential

Does your paper address subitem 6a-ii?

Copy and paste relevant sections from manuscript text

Your answer

6a-iii) Describe whether, how, and when qualitative feedback from participants was obtained

Describe whether, how, and when qualitative feedback from participants was obtained (e.g., through emails, feedback forms, interviews, focus groups).

	1	2	3	4	5	
subitem not at all important	○	○	○	◉	○	essential

Clear selection

Does your paper address subitem 6a-iii?

Copy and paste relevant sections from manuscript text

"For all groups, we assessed satisfaction with the mode of care delivery using surveys, which were administered to the patient and the physical therapist at the in-clinic discharge visit via the Research Electronic Data Capture (REDCap) platform.[18] Participating physical therapists responded after each care episode, considering the mode of delivery for that specific patient. Each survey assessed eight constructs; six satisfaction items were rated on a 5-point Likert scale (“very poor”, “poor”, “fair”, “good”, or “very good”), one item pertaining to safety used a 0 (“very unsafe”) to 100 (“very safe”) rating, and one item asked how likely the respondent would be to recommend their mode of delivery (for patient respondents) or use the same mode of delivery for a future similar patient (for therapist respondents). A final open-ended question sought any other written feedback."

6b) Any changes to trial outcomes after the trial commenced, with reasons

Does your paper address CONSORT subitem 6b? *

Copy and paste relevant sections from the manuscript (include quotes in quotation marks "like this" to indicate direct quotes from your manuscript), or elaborate on this item by providing additional information not in the ms, or briefly explain why the item is not applicable/relevant for your study

Trial outcomes did not change after the trial commenced.

7a) How sample size was determined

NPT: When applicable, details of whether and how the clustering by care provides or centers was addressed

7a-i) Describe whether and how expected attrition was taken into account when calculating the sample size

Describe whether and how expected attrition was taken into account when calculating the sample size.

	1	2	3	4	5	
subitem not at all important	(●)	○	○	○	○	essential

Clear selection

Does your paper address subitem 7a-i?

Copy and paste relevant sections from manuscript title (include quotes in quotation marks "like this" to indicate direct quotes from your manuscript), or elaborate on this item by providing additional information not in the ms, or briefly explain why the item is not applicable/relevant for your study

As a pilot feasibility study, sample size for statistical power was not estimated a priori.

7b) When applicable, explanation of any interim analyses and stopping guidelines

Does your paper address CONSORT subitem 7b? *

Copy and paste relevant sections from the manuscript (include quotes in quotation marks "like this" to indicate direct quotes from your manuscript), or elaborate on this item by providing additional information not in the ms, or briefly explain why the item is not applicable/relevant for your study

Not applicable.

Your answer must have a minimum of 25 characters.

8a) Method used to generate the random allocation sequence

NPT: When applicable, how care providers were allocated to each trial group

Does your paper address CONSORT subitem 8a? *

Copy and paste relevant sections from the manuscript (include quotes in quotation marks "like this" to indicate direct quotes from your manuscript), or elaborate on this item by providing additional information not in the ms, or briefly explain why the item is not applicable/relevant for your study

"Randomization was concealed and completed by a study coordinator using the REDCap randomization module.[18] Patients were 1:1 block randomized by site into one of three groups: a clinic+TR group, a TR-only group, and a usual care control group."

8b) Type of randomisation; details of any restriction (such as blocking and block size)

Does your paper address CONSORT subitem 8b? *

Copy and paste relevant sections from the manuscript (include quotes in quotation marks "like this" to indicate direct quotes from your manuscript), or elaborate on this item by providing additional information not in the ms, or briefly explain why the item is not applicable/relevant for your study

"Randomization was concealed and completed by a study coordinator using the REDCap randomization module.[18] Patients were 1:1 block randomized by site into one of three groups: a clinic+TR group, a TR-only group, and a usual care control group."

9) Mechanism used to implement the random allocation sequence (such as sequentially numbered containers), describing any steps taken to conceal the sequence until interventions were assigned

Does your paper address CONSORT subitem 9? *

Copy and paste relevant sections from the manuscript (include quotes in quotation marks "like this" to indicate direct quotes from your manuscript), or elaborate on this item by providing additional information not in the ms, or briefly explain why the item is not applicable/relevant for your study

"Randomization was concealed and completed by a study coordinator using the REDCap randomization module.[18] Patients were 1:1 block randomized by site into one of three groups: a clinic+TR group, a TR-only group, and a usual care control group."

10) Who generated the random allocation sequence, who enrolled participants, and who assigned participants to interventions

Does your paper address CONSORT subitem 10? *

Copy and paste relevant sections from the manuscript (include quotes in quotation marks "like this" to indicate direct quotes from your manuscript), or elaborate on this item by providing additional information not in the ms, or briefly explain why the item is not applicable/relevant for your study

"The randomization allocation sequence was generated by the PI. Randomization was concealed and completed by a study coordinator using the REDCap randomization module."

11a) If done, who was blinded after assignment to interventions (for example, participants, care providers, those assessing outcomes) and how

NPT: Whether or not administering co-interventions were blinded to group assignment

11a-i) Specify who was blinded, and who wasn't

Specify who was blinded, and who wasn't. Usually, in web-based trials it is not possible to blind the participants [1, 3] (this should be clearly acknowledged), but it may be possible to blind outcome assessors, those doing data analysis or those administering co-interventions (if any).

	1	2	3	4	5	
subitem not at all important	○	●	○	○	○	essential

Clear selection

Does your paper address subitem 11a-i? *

Copy and paste relevant sections from the manuscript (include quotes in quotation marks "like this" to indicate direct quotes from your manuscript), or elaborate on this item by providing additional information not in the ms, or briefly explain why the item is not applicable/relevant for your study

"This was an unblinded randomized controlled trial."

11a-ii) Discuss e.g., whether participants knew which intervention was the “intervention of interest” and which one was the “comparator”

Informed consent procedures (4a-ii) can create biases and certain expectations - discuss e.g., whether participants knew which intervention was the “intervention of interest” and which one was the “comparator”.

	1	2	3	4	5	
subitem not at all important	○	◉	○	○	○	essential

Clear selection

Does your paper address subitem 11a-ii?

Copy and paste relevant sections from the manuscript (include quotes in quotation marks "like this" to indicate direct quotes from your manuscript), or elaborate on this item by providing additional information not in the ms, or briefly explain why the item is not applicable/relevant for your study

"This was an unblinded randomized controlled trial."

11b) If relevant, description of the similarity of interventions

(this item is usually not relevant for ehealth trials as it refers to similarity of a placebo or sham intervention to a active medication/intervention)

Does your paper address CONSORT subitem 11b? *

Copy and paste relevant sections from the manuscript (include quotes in quotation marks "like this" to indicate direct quotes from your manuscript), or elaborate on this item by providing additional information not in the ms, or briefly explain why the item is not applicable/relevant for your study

Not applicable since this was pilot feasibility study without blinding.

12a) Statistical methods used to compare groups for primary and secondary outcomes

NPT: When applicable, details of whether and how the clustering by care providers or centers was addressed

Does your paper address CONSORT subitem 12a? *

Copy and paste relevant sections from the manuscript (include quotes in quotation marks "like this" to indicate direct quotes from your manuscript), or elaborate on this item by providing additional information not in the ms, or briefly explain why the item is not applicable/relevant for your study

Not applicable since this was a pilot feasibility study.

12a-i) Imputation techniques to deal with attrition / missing values

Imputation techniques to deal with attrition / missing values: Not all participants will use the intervention/comparator as intended and attrition is typically high in ehealth trials. Specify how participants who did not use the application or dropped out from the trial were treated in the statistical analysis (a complete case analysis is strongly discouraged, and simple imputation techniques such as LOCF may also be problematic [4]).

	1	2	3	4	5	
subitem not at all important	◉	○	○	○	○	essential

Clear selection

Does your paper address subitem 12a-i? *

Copy and paste relevant sections from the manuscript (include quotes in quotation marks "like this" to indicate direct quotes from your manuscript), or elaborate on this item by providing additional information not in the ms, or briefly explain why the item is not applicable/relevant for your study

Only one patient was withdrawn from the study, prior to beginning the intervention. There was no other attrition and we had complete data for other participants.

12b) Methods for additional analyses, such as subgroup analyses and adjusted analyses

Does your paper address CONSORT subitem 12b? *

Copy and paste relevant sections from the manuscript (include quotes in quotation marks "like this" to indicate direct quotes from your manuscript), or elaborate on this item by providing additional information not in the ms, or briefly explain why the item is not applicable/relevant for your study

Not applicable since this was a pilot feasibility study and therefore not adequately powered for a subgroup or adjusted analysis.

X26) REB/IRB Approval and Ethical Considerations [recommended as subheading under "Methods"] (not a CONSORT item)

X26-i) Comment on ethics committee approval

	1	2	3	4	5	
subitem not at all important	○	○	○	○	◉	essential

Clear selection

Does your paper address subitem X26-i?

Copy and paste relevant sections from the manuscript (include quotes in quotation marks "like this" to indicate direct quotes from your manuscript), or elaborate on this item by providing additional information not in the ms, or briefly explain why the item is not applicable/relevant for your study

"This study, including a valid primary informed consent process, was approved by the Cleveland Clinic Institutional Review Board (#19-544)."

x26-ii) Outline informed consent procedures

Outline informed consent procedures e.g., if consent was obtained offline or online (how? Checkbox, etc.?), and what information was provided (see 4a-ii). See [6] for some items to be included in informed consent documents.

	1	2	3	4	5	
subitem not at all important	○	○	○	◉	○	essential

Clear selection

Does your paper address subitem X26-ii?

Copy and paste relevant sections from the manuscript (include quotes in quotation marks "like this" to indicate direct quotes from your manuscript), or elaborate on this item by providing additional information not in the ms, or briefly explain why the item is not applicable/relevant for your study

"This study, including a valid primary informed consent process, was approved by the Cleveland Clinic Institutional Review Board (#19-544)."

X26-iii) Safety and security procedures

Safety and security procedures, incl. privacy considerations, and any steps taken to reduce the likelihood or detection of harm (e.g., education and training, availability of a hotline)

	1	2	3	4	5	
subitem not at all important	○	○	○	○	○	essential

Does your paper address subitem X26-iii?

Copy and paste relevant sections from the manuscript (include quotes in quotation marks "like this" to indicate direct quotes from your manuscript), or elaborate on this item by providing additional information not in the ms, or briefly explain why the item is not applicable/relevant for your study

"Additionally, the WizeCare platform enabled a safety assessment with each session in which a patient interacted with the system, including their unsupervised home exercise sessions. At the conclusion of each session on the platform, patients were asked, “Did you feel safe during this session?” If they answered “No”, the system sent an automated message to the treating physical therapist. The system also provided an automated response to the patient to, “Please stop participating in the exercise program and call your treating physical therapist.” Initiated by either the physical therapist or the patient within 24 hours of the reported lack of safety, a conversation between them allowed the patient to express any safety concerns. Any such events were recorded as adverse events, with subsequent steps as outlined above. These patient self-reports of safety were included amongst exploratory outcomes."

RESULTS

13a) For each group, the numbers of participants who were randomly assigned, received intended treatment, and were analysed for the primary outcome

NPT: The number of care providers or centers performing the intervention in each group and the number of patients treated by each care provider in each center

Does your paper address CONSORT subitem 13a? *

Copy and paste relevant sections from the manuscript (include quotes in quotation marks "like this" to indicate direct quotes from your manuscript), or elaborate on this item by providing additional information not in the ms, or briefly explain why the item is not applicable/relevant for your study

"In sum, 389 patients were screened for potential inclusion. Of these, 68 (17.5%) met inclusion criteria; 27 (39.7% of those eligible) were consented and screened in person (see CONSORT diagram in Figure 1). After 7 patients were excluded at the in-person screening, 20 were enrolled and randomized to the Clinic+TR group (N=6), the TR-only group (N=6), and to the usual care group (N=8)."

"Of the 19 who completed the study, 12 were enrolled in Las Vegas and 7 were enrolled in Cleveland. There were 7 enrolled for a new episode of physical therapy while 12 were enrolled as part of an established episode. Five physical therapists (2 in Las Vegas and 3 in Cleveland) provided the interventions, with each episode managed by only one physical therapist."

13b) For each group, losses and exclusions after randomisation, together with reasons

Does your paper address CONSORT subitem 13b? (NOTE: Preferably, this is shown in a CONSORT flow diagram) *

Copy and paste relevant sections from the manuscript (include quotes in quotation marks "like this" to indicate direct quotes from your manuscript), or elaborate on this item by providing additional information not in the ms, or briefly explain why the item is not applicable/relevant for your study

"In sum, 389 patients were screened for potential inclusion. Of these, 68 (17.5%) met inclusion criteria; 27 (39.7% of those eligible) were consented and screened in person (see CONSORT diagram in Figure 1). After 7 patients were excluded at the in-person screening, 20 were enrolled and randomized to the Clinic+TR group (N=6), the TR-only group (N=6), and to the usual care group (N=8). One patient (TR-only group) was withdrawn from the study after a non-study related injurious fall that resulted in a hospitalization. Two other patients (both in usual care) reported non-study related non-injurious falls and remained in the study."

13b-i) Attrition diagram

Strongly recommended: An attrition diagram (e.g., proportion of participants still logging in or using the intervention/comparator in each group plotted over time, similar to a survival curve) or other figures or tables demonstrating usage/dose/engagement.

	1	2	3	4	5	
subitem not at all important	◉	○	○	○	○	essential

Clear selection

Does your paper address subitem 13b-i?

Copy and paste relevant sections from the manuscript or cite the figure number if applicable (include quotes in quotation marks "like this" to indicate direct quotes from your manuscript), or elaborate on this item by providing additional information not in the ms, or briefly explain why the item is not applicable/relevant for your study

Not addressed since this was a short-term pilot feasibility study.

14a) Dates defining the periods of recruitment and follow-up

Does your paper address CONSORT subitem 14a? *

Copy and paste relevant sections from the manuscript (include quotes in quotation marks "like this" to indicate direct quotes from your manuscript), or elaborate on this item by providing additional information not in the ms, or briefly explain why the item is not applicable/relevant for your study

"Patients were recruited between February 1, 2020 and December 15, 2022. Initially, patients were identified for pre-screening if they had a diagnosis of PD for any duration and were scheduled for a new episode of physical therapy."

14a-i) Indicate if critical “secular events” fell into the study period

Indicate if critical “secular events” fell into the study period, e.g., significant changes in Internet resources available or “changes in computer hardware or Internet delivery resources”

	1	2	3	4	5	
subitem not at all important	(•)	( )	( )	( )	( )	essential

Clear selection

Does your paper address subitem 14a-i?

Copy and paste relevant sections from the manuscript (include quotes in quotation marks "like this" to indicate direct quotes from your manuscript), or elaborate on this item by providing additional information not in the ms, or briefly explain why the item is not applicable/relevant for your study

Not addressed; not a concern for this study.

14b) Why the trial ended or was stopped (early)

Does your paper address CONSORT subitem 14b? *

Copy and paste relevant sections from the manuscript (include quotes in quotation marks "like this" to indicate direct quotes from your manuscript), or elaborate on this item by providing additional information not in the ms, or briefly explain why the item is not applicable/relevant for your study

"Due to slowed enrollment throughout the COVID-19 pandemic, the target was decreased to 20 patients in conjunction with the April 2022 amendment described above."

15) A table showing baseline demographic and clinical characteristics for each group

NPT: When applicable, a description of care providers (case volume, qualification, expertise, etc.) and centers (volume) in each group

Does your paper address CONSORT subitem 15? *

Copy and paste relevant sections from the manuscript (include quotes in quotation marks "like this" to indicate direct quotes from your manuscript), or elaborate on this item by providing additional information not in the ms, or briefly explain why the item is not applicable/relevant for your study

"Baseline participant characteristics are shown in Table 1. There were no statistical differences between groups; however, potentially meaningful clinical differences were noted in years since PD diagnosis (median [IQR] = 5 [1-8] for the Clinic+TR group compared to 2 [0-3] for the TR-only and usual care groups). Similarly, other baseline markers (MDS-UPDRS III score, Hoehn & Yahr stage, PDQ summary index, and ABC score) suggested advanced disease progression among those in the Clinic+TR group compared to other groups."

15-i) Report demographics associated with digital divide issues

In ehealth trials it is particularly important to report demographics associated with digital divide issues, such as age, education, gender, social-economic status, computer/Internet/ehealth literacy of the participants, if known.

	1	2	3	4	5	
subitem not at all important	(●)	○	○	○	○	essential

Clear selection

Does your paper address subitem 15-i? *

Copy and paste relevant sections from the manuscript (include quotes in quotation marks "like this" to indicate direct quotes from your manuscript), or elaborate on this item by providing additional information not in the ms, or briefly explain why the item is not applicable/relevant for your study

This was not explicitly assessed. Though we did report in Table 1 that 9 out of 68 eligible patients could not be enrolled due to not having reliable internet in their home.

16) For each group, number of participants (denominator) included in each analysis and whether the analysis was by original assigned groups

16-i) Report multiple “denominators” and provide definitions

Report multiple “denominators” and provide definitions: Report N’s (and effect sizes) “across a range of study participation [and use] thresholds” [1], e.g., N exposed, N consented, N used more than x times, N used more than y weeks, N participants “used” the intervention/comparator at specific pre-defined time points of interest (in absolute and relative numbers per group). Always clearly define “use” of the intervention.

	1	2	3	4	5	
subitem not at all important	○	◉	○	○	○	essential

Clear selection

Does your paper address subitem 16-i? *

Copy and paste relevant sections from the manuscript (include quotes in quotation marks "like this" to indicate direct quotes from your manuscript), or elaborate on this item by providing additional information not in the ms, or briefly explain why the item is not applicable/relevant for your study

Use was assessed qualitatively in this pilot feasibility study.

16-ii) Primary analysis should be intent-to-treat

Primary analysis should be intent-to-treat, secondary analyses could include comparing only “users”, with the appropriate caveats that this is no longer a randomized sample (see 18-i).

	1	2	3	4	5	
subitem not at all important	○	○	◉	○	○	essential

Clear selection

Does your paper address subitem 16-ii?

Copy and paste relevant sections from the manuscript (include quotes in quotation marks "like this" to indicate direct quotes from your manuscript), or elaborate on this item by providing additional information not in the ms, or briefly explain why the item is not applicable/relevant for your study

Your answer

17a) For each primary and secondary outcome, results for each group, and the estimated effect size and its precision (such as 95% confidence interval)

Does your paper address CONSORT subitem 17a? *

Copy and paste relevant sections from the manuscript (include quotes in quotation marks "like this" to indicate direct quotes from your manuscript), or elaborate on this item by providing additional information not in the ms, or briefly explain why the item is not applicable/relevant for your study

"Between all three groups, there were no statistically significant differences in clinical outcomes at the conclusion of the physical therapy episode. In pair-wise group comparisons, there was a difference in final ABC between those in the Clinic+TR group (mean=57.7±21.4) and the usual care group (mean=79.4±11.4; P=.03). However, there was no within-group difference for the ABC for any group. We observed statistically significant improvements from baseline to final assessment in the usual care group for the 5STS time (median [IQR]=10.1 [9.2-12.8] seconds at baseline and 9.2 [8.8-11.3] seconds at final; P=.04) and the MiniBESTest score (mean=20.5±3.3 at baseline and 23.0±2.4 at final; P<.01). The Clinic+TR group also improved in MiniBESTest score from baseline to final (mean=19.8±2.3 at baseline and 22.2±2.2 at final; P=.02). Between-group comparison are in Table 2; within-group comparisons are in Table 3."

17a-i) Presentation of process outcomes such as metrics of use and intensity of use

In addition to primary/secondary (clinical) outcomes, the presentation of process outcomes such as metrics of use and intensity of use (dose, exposure) and their operational definitions is critical. This does not only refer to metrics of attrition (13-b) (often a binary variable), but also to more continuous exposure metrics such as “average session length”. These must be accompanied by a technical description how a metric like a “session” is defined (e.g., timeout after idle time) [1] (report under item 6a).

	1	2	3	4	5	
subitem not at all important	○	◉	○	○	○	essential

Clear selection

Does your paper address subitem 17a-i?

Copy and paste relevant sections from the manuscript (include quotes in quotation marks "like this" to indicate direct quotes from your manuscript), or elaborate on this item by providing additional information not in the ms, or briefly explain why the item is not applicable/relevant for your study

Use was assessed qualitatively in this pilot feasibility study.

17b) For binary outcomes, presentation of both absolute and relative effect sizes is recommended

Does your paper address CONSORT subitem 17b? *

Copy and paste relevant sections from the manuscript (include quotes in quotation marks "like this" to indicate direct quotes from your manuscript), or elaborate on this item by providing additional information not in the ms, or briefly explain why the item is not applicable/relevant for your study

Not applicable. We did not have binary outcomes.

18) Results of any other analyses performed, including subgroup analyses and adjusted analyses, distinguishing pre-specified from exploratory

Does your paper address CONSORT subitem 18? *

Copy and paste relevant sections from the manuscript (include quotes in quotation marks "like this" to indicate direct quotes from your manuscript), or elaborate on this item by providing additional information not in the ms, or briefly explain why the item is not applicable/relevant for your study

"For the primary analysis of feasibility, Fisher's exact tests were used to compare patient and physical therapist responses to the six survey items of care delivery satisfaction and the single question assessing recommendation for future care."

"For exploratory analyses, the between-group differences in discharge clinical outcomes were compared using ANOVA for normally distributed data (TUG time, TUG-Cognitive time, 10-m walk test, 6MWT distance, Mini-BESTest, and ABC) or Kruskal-Wallis tests for skewed data (PDQ-39 and 5STS)."

18-i) Subgroup analysis of comparing only users

A subgroup analysis of comparing only users is not uncommon in ehealth trials, but if done, it must be stressed that this is a self-selected sample and no longer an unbiased sample from a randomized trial (see 16-iii).

	1	2	3	4	5	
subitem not at all important	(selected)	○	○	○	○	essential

Clear selection

Does your paper address subitem 18-i?

Copy and paste relevant sections from the manuscript (include quotes in quotation marks "like this" to indicate direct quotes from your manuscript), or elaborate on this item by providing additional information not in the ms, or briefly explain why the item is not applicable/relevant for your study

Not applicable. All participants used the platform.

19) All important harms or unintended effects in each group
(for specific guidance see CONSORT for harms)

Does your paper address CONSORT subitem 19? *

Copy and paste relevant sections from the manuscript (include quotes in quotation marks "like this" to indicate direct quotes from your manuscript), or elaborate on this item by providing additional information not in the ms, or briefly explain why the item is not applicable/relevant for your study

"One patient (TR-only group) was withdrawn from the study after a non-study related injurious fall that resulted in a hospitalization. Two other patients (both in usual care) reported non-study related non-injurious falls and remained in the study. There were 0 instances in which patients reported feeling unsafe after a TR session."

19-i) Include privacy breaches, technical problems

Include privacy breaches, technical problems. This does not only include physical "harm" to participants, but also incidents such as perceived or real privacy breaches [1], technical problems, and other unexpected/unintended incidents. "Unintended effects" also includes unintended positive effects [2].

	1	2	3	4	5	
subitem not at all important	◉	○	○	○	○	essential

Clear selection

Does your paper address subitem 19-i?

Copy and paste relevant sections from the manuscript (include quotes in quotation marks "like this" to indicate direct quotes from your manuscript), or elaborate on this item by providing additional information not in the ms, or briefly explain why the item is not applicable/relevant for your study

Not applicable; none to report.

19-ii) Include qualitative feedback from participants or observations from staff/researchers

Include qualitative feedback from participants or observations from staff/researchers, if available, on strengths and shortcomings of the application, especially if they point to unintended/unexpected effects or uses. This includes (if available) reasons for why people did or did not use the application as intended by the developers.

	1	2	3	4	5	
subitem not at all important	○	○	○	◉	○	essential

Clear selection

Does your paper address subitem 19-ii?

Copy and paste relevant sections from the manuscript (include quotes in quotation marks "like this" to indicate direct quotes from your manuscript), or elaborate on this item by providing additional information not in the ms, or briefly explain why the item is not applicable/relevant for your study

"As shown in Figure 2, patients in all groups generally rated their care delivery as “good” or “very good” across all constructs assessed, including their overall satisfaction. No differences were statistically significant. However, fewer patients in both the Clinic+TR and TR-only groups (50% and 40%, respectively) gave a rating of “very good” than in the usual care group (75%) when asked if the prescribed exercises met their needs. Patients’ rating of safety was high across all groups (median [IQR] = 81.5 [72-96] for Clinic+TR, 100 [100-100] for TR-only, and 98 [82.5-100] for usual care); these ratings did significantly differ between groups (P=0.049). Patients in all groups generally said they would “very likely” recommend their course of physical therapy for another patient with PD; exceptions were one patient in the Clinic+TR group reporting “somewhat unlikely,” one patient in the TR-only group reporting “very unlikely,” and one patient in each of the TR-only group and usual care group reporting “somewhat likely.”

"Among the five physical therapists who participated as care providers, all five treated patients in the usual care group, four treated patients in the Clinic+TR group, and three treated patients in the TR-only group. In rating their experience of the provision of care for all 19 patients who completed the study, the therapists’ responses were varied (Figure 3). Between groups, there was a statistical difference (P=.03) in therapists’ ratings in the ability to provide instructions to patients regarding prescribed exercises. Fewer therapists rated “Very good” for the TR-only group (20.0%) than for the usual care group (87.5%). No other differences in ratings were statistically significant. Therapists’ rating of safety for patients was high across all groups (median [IQR] = 91.5 [71-100] for Clinic+TR, 85 [82-88] for TR-only, and 90 [81-99] for usual care); these ratings did not differ significantly between groups (P=.81). Therapists’ responses were varied in regards to the likelihood that they would recommend a similar course of treatment for a patient with PD presenting with similar characteristics. Though not statistically significant (P=.08), the ratings for “Very likely” were 66.7% for the Clinic+TR group, 0% for the TR-only group, and 62.5% for the usual care group."

DISCUSSION

22) Interpretation consistent with results, balancing benefits and harms, and considering other relevant evidence

NPT: In addition, take into account the choice of the comparator, lack of or partial blinding, and unequal expertise of care providers or centers in each group

22-i) Restate study questions and summarize the answers suggested by the data, starting with primary outcomes and process outcomes (use)

Restate study questions and summarize the answers suggested by the data, starting with primary outcomes and process outcomes (use).

	1	2	3	4	5	
subitem not at all important	○	○	○	◉	○	essential

Clear selection

Does your paper address subitem 22-i? *

Copy and paste relevant sections from the manuscript (include quotes in quotation marks "like this" to indicate direct quotes from your manuscript), or elaborate on this item by providing additional information not in the ms, or briefly explain why the item is not applicable/relevant for your study

"In this pilot randomized clinical trial of telerehabilitation for patients with Parkinson's disease, we screened 389 patient records for potential eligibility, included 20 patients after a series of exclusions, and analyzed data on satisfaction and clinical outcomes for 19 patients. In general, TR was found to be feasible and an accepted mode of therapy administration by both patients and therapists."

22-ii) Highlight unanswered new questions, suggest future research

Highlight unanswered new questions, suggest future research.

	1	2	3	4	5	
subitem not at all important	○	○	○	◉	○	essential

Clear selection

Does your paper address subitem 22-ii?

Copy and paste relevant sections from the manuscript (include quotes in quotation marks "like this" to indicate direct quotes from your manuscript), or elaborate on this item by providing additional information not in the ms, or briefly explain why the item is not applicable/relevant for your study

"Some individuals who met clinical criteria for participation did not have reliable internet and were therefore excluded. Future studies that provide WIFI as needed would ensure that underserved populations are equally eligible and represented for TR interventions."

"Though intentional for this pilot trial, our small sample size means that the results are not generalizable to a larger population of similar patients with PD. These issues with recruitment and sampling need to be addressed in a larger trial."

"An optimal care delivery strategy might include shared decision-making with patients so that TR is combined appropriately with in-person visits as part of the rehabilitation episode. This requires testing in future trials. In a future larger trial, eligibility criteria should be such that patients have similar recent experience with rehabilitation interventions and the availability of therapists to provide the intervention needs to be assured so that a truly random sample can be recruited."

20) Trial limitations, addressing sources of potential bias, imprecision, and, if relevant, multiplicity of analyses

20-i) Typical limitations in ehealth trials

Typical limitations in ehealth trials: Participants in ehealth trials are rarely blinded. Ehealth trials often look at a multiplicity of outcomes, increasing risk for a Type I error. Discuss biases due to non-use of the intervention/usability issues, biases through informed consent procedures, unexpected events.

	1	2	3	4	5	
subitem not at all important	○	○	○	◉	○	essential

Clear selection

Does your paper address subitem 20-i? *

Copy and paste relevant sections from the manuscript (include quotes in quotation marks "like this" to indicate direct quotes from your manuscript), or elaborate on this item by providing additional information not in the ms, or briefly explain why the item is not applicable/relevant for your study

"Since recruitment success was a primary outcome, protocol amendments that changed the eligibility criteria at two different points in the study influenced this outcome. In conjunction, these changes altered the characteristics of participants (12 who were continuing an active episode of therapy vs. 7 who were starting a new episode) in ways that introduce bias that could not reliably be measured. Similarly, our sampling method was neither random nor consecutive since nearly 25% of patients who were clinically eligible at initial screening simply could not be scheduled for a visit due to a lack of therapist availability."

"Clinical outcomes were obtained by the study coordinator at baseline and the treating therapists at post-intervention, which may impact measurement reliability. Additionally, the therapist completing post-intervention clinical testing was not blinded to group allocation, potentially introducing bias."

21) Generalisability (external validity, applicability) of the trial findings

NPT: External validity of the trial findings according to the intervention, comparators, patients, and care providers or centers involved in the trial

21-i) Generalizability to other populations

Generalizability to other populations: In particular, discuss generalizability to a general Internet population, outside of a RCT setting, and general patient population, including applicability of the study results for other organizations

	1	2	3	4	5	
subitem not at all important	○	○	○	◉	○	essential

Clear selection

Does your paper address subitem 21-i?

Copy and paste relevant sections from the manuscript (include quotes in quotation marks "like this" to indicate direct quotes from your manuscript), or elaborate on this item by providing additional information not in the ms, or briefly explain why the item is not applicable/relevant for your study

"The survey responses (including the comments shown in Appendix 2) highlight the potential for matching patients to the optimal utilization of a TR platform as part of the therapy episode. A few patients and therapists commented in some instances that a specific approach (i.e., in-person, TR only, or a hybrid model) would be preferred for certain patients. While our study design did not allow for cross-over outside of allocated group, it would be reasonable to consider clinical models in which a patient's presentation (e.g., type and severity of deficits, distance lived from the clinic, comfort with technology, presence and ability of at-home caregivers, etc.) could guide the use of TR. In such cases, shared decision-making would be an appropriate approach to planning.[29], [30]"

21-ii) Discuss if there were elements in the RCT that would be different in a routine application setting

Discuss if there were elements in the RCT that would be different in a routine application setting (e.g., prompts/reminders, more human involvement, training sessions or other co-interventions) and what impact the omission of these elements could have on use, adoption, or outcomes if the intervention is applied outside of a RCT setting.

	1	2	3	4	5	
subitem not at all important	○	○	○	◉	○	essential

Clear selection

Does your paper address subitem 21-ii?

Copy and paste relevant sections from the manuscript (include quotes in quotation marks "like this" to indicate direct quotes from your manuscript), or elaborate on this item by providing additional information not in the ms, or briefly explain why the item is not applicable/relevant for your study

"The survey responses (including the comments shown in Appendix 2) highlight the potential for matching patients to the optimal utilization of a TR platform as part of the therapy episode. A few patients and therapists commented in some instances that a specific approach (i.e., in-person, TR only, or a hybrid model) would be preferred for certain patients. While our study design did not allow for cross-over outside of allocated group, it would be reasonable to consider clinical models in which a patient's presentation (e.g., type and severity of deficits, distance lived from the clinic, comfort with technology, presence and ability of at-home caregivers, etc.) could guide the use of TR. In such cases, shared decision-making would be an appropriate approach to planning.[29], [30]"

OTHER INFORMATION

23) Registration number and name of trial registry

Does your paper address CONSORT subitem 23? *

Copy and paste relevant sections from the manuscript (include quotes in quotation marks "like this" to indicate direct quotes from your manuscript), or elaborate on this item by providing additional information not in the ms, or briefly explain why the item is not applicable/relevant for your study

The study was retrospectively registered in ClinicalTrials.gov (NCT06246747) since prospective registration was not deemed essential for this pilot study.

24) Where the full trial protocol can be accessed, if available

Does your paper address CONSORT subitem 24? *

Cite a Multimedia Appendix, other reference, or copy and paste relevant sections from the manuscript (include quotes in quotation marks "like this" to indicate direct quotes from your manuscript), or elaborate on this item by providing additional information not in the ms, or briefly explain why the item is not applicable/relevant for your study

A full trial protocol was not prepared for publication. Readers can access the protocol details at ClinicalTrials.gov.

25) Sources of funding and other support (such as supply of drugs), role of funders

Does your paper address CONSORT subitem 25? *

Copy and paste relevant sections from the manuscript (include quotes in quotation marks "like this" to indicate direct quotes from your manuscript), or elaborate on this item by providing additional information not in the ms, or briefly explain why the item is not applicable/relevant for your study

"WizeCare Technologies (Or Yehuda, Israel) funded this study. The sponsor provided mobile tablets as an option for patient use. Otherwise, WizeCare played no role in the design, execution, or reporting of the study."

X27) Conflicts of Interest (not a CONSORT item)

X27-i) State the relation of the study team towards the system being evaluated

In addition to the usual declaration of interests (financial or otherwise), also state the relation of the study team towards the system being evaluated, i.e., state if the authors/evaluators are distinct from or identical with the developers/sponsors of the intervention.

	1	2	3	4	5	
subitem not at all important	○	○	◉	○	○	essential

Clear selection

Does your paper address subitem X27-i?

Copy and paste relevant sections from the manuscript (include quotes in quotation marks "like this" to indicate direct quotes from your manuscript), or elaborate on this item by providing additional information not in the ms, or briefly explain why the item is not applicable/relevant for your study

"None of the investigators have any affiliation with WizeCare Technologies."

About the CONSORT EHEALTH checklist

As a result of using this checklist, did you make changes in your manuscript? *

- ○ yes, major changes
- ◉ yes, minor changes
- ○ no

What were the most important changes you made as a result of using this checklist?

Added the statements:

"No updates were incorporated to the TR platform during the conduct of this trial."
"None of the investigators have any affiliation with WizeCare Technologies."

How much time did you spend on going through the checklist INCLUDING making changes in your manuscript *

Approximately 60 minutes spent

As a result of using this checklist, do you think your manuscript has improved? *

- (selected) yes
- no
- Other:

Would you like to become involved in the CONSORT EHEALTH group?

This would involve for example becoming involved in participating in a workshop and writing an "Explanation and Elaboration" document

- yes
- (selected) no
- Other:

Clear selection

Any other comments or questions on CONSORT EHEALTH

Your answer

STOP - Save this form as PDF before you click submit

To generate a record that you filled in this form, we recommend to generate a PDF of this page (on a Mac, simply select "print" and then select "print as PDF") before you submit it.

When you submit your (revised) paper to JMIR, please upload the PDF as supplementary file.

Don't worry if some text in the textboxes is cut off, as we still have the complete information in our database. Thank you!

Final step: Click submit !

Click submit so we have your answers in our database!

Submit

Clear form

Never submit passwords through Google Forms.

This content is neither created nor endorsed by Google. Report Abuse - Terms of Service - Privacy Policy

Google Forms
